# Time-resolved serial synchrotron and serial femtosecond crystallography of heme proteins using photocaged nitric oxide

**DOI:** 10.1107/S2052252525006645

**Published:** 2025-08-22

**Authors:** Peter Smyth, Sofia Jaho, Lewis J. Williams, Gabriel Karras, Ann Fitzpatrick, Amy J. Thompson, Sinan Battah, Danny Axford, Sam Horrell, Marina Lučić, Kotone Ishihara, Machika Kataoka, Hiroaki Matsuura, Kanji Shimba, Kensuke Tono, Takehiko Tosha, Hiroshi Sugimoto, Shigeki Owada, Michael A. Hough, Jonathan A.R. Worrall, Robin L. Owen

**Affiliations:** a Diamond Light Source Harwell Science and Innovation Campus DidcotOX11 0DE United Kingdom; b School of Life Sciences University of Essex Wivenhoe Park Colchester EssexCO4 3SQ United Kingdom; c Research Complex at Harwell Rutherford Appleton Laboratory DidcotOX11 0FA United Kingdom; d University of Hyogo 3-2-1 Kouto Kamigori, Ako Hyogo678-1297 Japan; e RIKEN SPring-8 Center, 1-1-1 Kouto, Sayo, Hyogo679-5148, Japan; f Japan Synchrotron Radiation Research Institute 1-1-1 Kouto Sayo Hyogo679-5198 Japan; European Molecular Biology Laboratory, France

**Keywords:** photocages, heme proteins, serial crystallography, time-resolved crystallography, nitric oxide

## Abstract

Time-resolved X-ray crystallography is undergoing a renaissance due to the development of serial crystallography at synchrotron and XFEL beamlines. Crucial to such experiments are efficient and effective methods for uniformly initiating time-dependent processes within microcrystals, such as ligand binding, enzymatic reactions or signalling. A widely applicable approach is the use of photocaged substrates, where the photocage is soaked into the crystal in advance and then activated using a laser pulse to provide uniform initiation of the reaction throughout the crystal. This work characterizes photocage release of nitric oxide and binding of this ligand to two heme protein systems, cytochrome *c*′-β and dye-decolourizing peroxidase B using a fixed target sample delivery system. Laser parameters for photoactivation are systematically explored, and time-resolved structures over timescales ranging from 100 µs to 1.4 s using synchrotron and XFEL beamlines are described. The effective use of this photocage for time-resolved crystallography is demonstrated and appropriate illumination conditions for such experiments are determined.

## Abstract 2

We demonstrate the application of a nitric oxide releasing photocage system for time-resolved serial crystallography studies of two heme containing proteins using a fixed target sample delivery system. Optimal parameters for successful photocage activation and nitric oxide release are explored.

## 1. Introduction

Determining time-resolved, transient, structures of proteins represents a grand challenge in structural biology with the promise to fully characterize enzyme reactions, ligand binding and signalling processes (Caramello & Royant, 2024 ▸; Poddar *et al.*, 2022 ▸). Realization of this goal has been significantly advanced by the development of serial crystallography, with reaction initiation, by various means, being followed by measurement of a single still diffraction image after a defined time delay. By varying the time delay, a stop-motion series of structures can be produced that illustrate the biological process under question, for example the catalytic reaction of an enzyme. The primary means of initiating reactions have been using light pulses or mixing microcrystals with a substrate or other reagents, although additional methods such as abrupt (laser-induced) temperature jumps are under development (Wolff *et al.*, 2023 ▸).

Reaction initiation using light pulses is a powerful approach enabling very fast timepoints to be probed in naturally photoactivatable systems such as photosystems, flavins or rhodopsins (Kupitz *et al.*, 2014 ▸; Nango *et al.*, 2016 ▸; Christou *et al.*, 2023 ▸). However, the vast majority of enzymatic reactions cannot be naturally activated by light (Monteiro *et al.*, 2021 ▸). Substrate-driven reactions can be probed using mixing approaches for reaction initiation, but such approaches are limited to timepoints in the tens of milliseconds range or greater due to the rate of diffusion of the substrate through the crystal, and thus the degree of synchronization of reaction initiation (Schmidt, 2020 ▸). The data described by Pandey *et al.* (2021 ▸) indicate that a 12-fold reduction in ligand diffusion coefficient (and thus an approximate 12-fold increase in diffusion time to the centre of a crystal), factoring in the effect of mother liquor and the crystal lattice on diffusion rates, gives good agreement between observed ligand occupancies and calculated ligand concentrations suggesting that timescales calculated in Schmidt (2020 ▸) might provide a best-case lower bound.

An approach that has attracted considerable interest is the use of photocages, where light releases a molecule of interest from a larger caged molecule that has been pre-soaked into the crystal (Monteiro *et al.*, 2021 ▸; Brändén & Neutze, 2021 ▸). Although thorough characterization of the photocage of interest – including decaging rate, optical properties, *in crystallo* stability and quantum yield – is required, photocages can provide a convenient means of accessing time-resolved substrate-driven serial crystallography (Monteiro *et al.*, 2021 ▸). Faster timepoints are more accessible with photocages than using mixing methods because the caged compound is released throughout the crystal effectively simultaneously. An alternative approach has been to photocage the protein itself with light activation allowing the protein to adopt an active conformation (Mangubat-Medina & Ball, 2021 ▸).

Previously a photocage releasing nitric oxide (NO) was used to access several intermediate states of cytochrome P450nor (Tosha *et al.*, 2017 ▸). A 20 ms timepoint was accessed using room-temperature SFX and a viscous media extruder, while a longer timepoint required rapid freeze quench of the microcrystals after photocage release. While this work demonstrated the effectiveness of the NO cage in time-resolved SFX, only short timepoints could be accessed at room temperature and only a single timepoint was obtained in each experiment. Our approach aims to establish a method enabling a very wide range of timepoints to be accessed at room temperature using the sample efficient fixed target approach and to do this at both synchrotron and XFEL sources. Another example of photocage based time-resolved serial crystallography includes characterization of allostery in fluoro­acetate dehalogenase over the timescales of 30 ms to 30 s using serial synchrotron crystallography (SSX) (Mehrabi *et al.*, 2019 ▸). Here, the photocaged fluoro­acetate was released with a pulse of 344 nm light: initial formation of the enzyme–substrate complex was followed by downstream catalysis. A further example is the use of photocaged zinc ions to initiate reactivity of a beta-lactamase (Wilamowski *et al.*, 2022 ▸), where 347 nm illumination was used to release zinc ions within microcrystals resulting in cleavage of the beta-lactam ring by the enzyme.

In this work, we describe a thorough characterization of the application of the NO photocage *N*,*N*′-bis-(carb­oxy­methyl)-*N*,*N*′-di­nitroso-1,4-phenyl­enedi­amine (henceforth referred to as NO cage) to room-temperature microcrystals of two proteins: a gas binding cytochrome *c*′ (McCP-β) and a dye-decolourizing peroxidase B (DtpB). McCP-β provides an excellent test case for the use of NO cage for two reasons. Firstly, upon NO binding, a vacant coordination position on the distal face of the heme iron becomes occupied, resulting in unambiguous electron density changes. Secondly, McCP-β provides a stepping-stone towards future time-resolved experiments with other cytochrome *c*′ proteins where the distal six-coordinate NO may only transiently exist as a structurally uncharacterized intermediate on the pathway to a five-coordinate NO complex on the proximal side of the heme. This provides a structural model for similar NO binding behaviour in the mammalian NO sensor soluble guanylate cyclase (Hough *et al.*, 2011 ▸; Hough & Andrew, 2015 ▸). DtpB is a hexameric heme peroxidase from *Streptomyces lividans* where each monomer contains a single five-coordinate heme in the ferric state (Lučić *et al.*, 2020 ▸; Lučić *et al.*, 2024 ▸). The distal heme pocket is unusual for a peroxidase in that it is devoid of water molecules (*i.e.* is a ‘dry pocket’) which allows a straightforward binding of NO to form a six-coordinate heme complex.

While our focus in this work has been to characterize applications of NO cage to suitable test proteins, it is of interest to consider the role of NO within the organisms in which these proteins are found. McCP-β occurs within the methano­trophic *Methyl­ococcus capsulatus* (Bath) together with a homologous cytochrome P460 enzyme that oxidizes hydroxyl­amine with NO as a reaction intermediate (Adams *et al.*, 2025 ▸). Possible roles for McCP-β include NO buffering to mediate toxicity/stress or transport between different enzymes that interact with NO. The functional relevance of NO binding to DtpB is not known, although potential roles for NO have been proposed within *S. lividans* including as a regulatory ligand for WhiB and other Wbl transcription factors (Chandra & Chater, 2014 ▸). DtpB resides in the cytoplasm of *S. lividans* and, while is generally considered to be a heme peroxidase, the precise physiological function has not yet been established. DtpB has low peroxidase activity with hydrogen peroxide but forms a long-lived (*t*_1/2_ > 1 h) two-electron oxidized catalytic intermediate known as compound I (Lučić *et al.*, 2020 ▸). A recent study highlighted that DtpB may act to ‘safely’ store oxidizing equivalents generated by the cell until a reducing substrate becomes available (Lučić *et al.*, 2024 ▸). The low peroxidase activity and ability to bind to NO implies that NO binding could also occur *in vivo*, particularly in cases of NO stress. A recent study in the close homolog *Streptomyces coelicolor* suggested that proteins related to NO sensing are present (Yoshizumi *et al.*, 2023 ▸). Intriguingly, addition of NO donor compounds to cell cultures promoted antibiotic production and regulated differentiation, suggesting that NO signalling is important for antibiotic production and morphological differentiation.

We describe an investigation of the most appropriate laser parameters for efficient activation of NO cage to achieve high-occupancy NO-bound states of both proteins and compare fixed target and high viscosity extruder sample delivery methods for photocage experiments. Once photocage release had been established, we explored several different timepoints, revealing differences in NO binding to the different heme centres in the hexameric DtpB. We demonstrate the applicability of this approach to time-resolved work across the microsecond to second time regimes.

## 2. Materials and methods

### 2.1. NO cage

The NO releasing photocage – *N*,*N*′-bis-(carb­oxy­methyl)-*N*,*N*′-di­nitroso-1,4-phenyl­enedi­amine (Namiki *et al.*, 1997 ▸; Cabail *et al.*, 2002 ▸) – was synthesized as described previously, in batches of 400 mg using a slightly adapted version of the published protocol [full details provided in Section S1 and Fig. S1(*a*) of the supporting information]. Upon excitation by UV light at ∼300 nm, where the extinction coefficient is highest (Section S1), the NO cage undergoes photolysis to release two NO molecules per photocage molecule [Fig. S1(*b*)]. The two NO molecules are released in a stepwise manner, with the first released within a microsecond and the second in tens of microseconds (Namiki *et al.*, 1997 ▸). The quantum yield of this reaction has previously been determined to be 1.4 (Tosha *et al.*, 2017 ▸).

### 2.2. McCP-β sample preparation

*Methyl­ococcus capsulatus* cytochrome *c*′-β (McCP-β) was expressed and purified as described previously (Adams *et al.*, 2019 ▸). Purified protein was buffer exchanged into 50 m*M* HEPES pH 7.5 and concentrated to 40 mg ml^−1^ (extinction coefficient ɛ_400_ ≃ 70 m*M*^−1^ cm^−1^). Batch microcrystallization was performed by mixing an equal volume of protein solution and crystallization buffer consisting of 70%(*v*/*v*) PEG 550, 100 m*M* MES pH 6.5 and 10 m*M* ZnSO_4_. Cubic crystals 30 µm across grew within 24 h when incubated at 18°C [Fig. S2(*a*)].

Prior to laser experiments, the NO cage was dissolved to 10 mg ml^−1^ in a separate mother liquor solution consisting of 25 m*M* HEPES pH 7.5, 35%(*v*/*v*) PEG 550, 50 m*M* MES pH 6.5 and 5 m*M* ZnSO_4_. This aimed to replicate the conditions the crystals were in, preventing crystal degradation upon mixing with photocage. Crystals were centrifuged briefly at 2000*g* and the supernatant was removed. The much smaller volume of concentrated crystals was then gently resuspended in 200 µl of photocage solution and soaked for 10 min. The high ratio of photocage solution to crystal volume kept the final photocage concentration close to the original 10 mg ml^−1^. Photocage preparation and subsequent chip loading were performed under red light, and data collection was performed on darkened beamlines to prevent premature NO release.

A structure of McCP-β with full NO binding was collected by pre-soaking crystals with a pH-dependent NO donor, 1-(hy­droxy-NNO-az­oxy)-l-proline. This compound, known as PROLI NONOate, dissociates into proline and two NO molecules in a pH-dependent manner (Saavedra *et al.*, 1996 ▸). The donor was dissolved at 1 mg ml^−1^ in 10 m*M* NaOH, then 30 µl was soaked into 180 µl of crystal slurry over 10 min, prior to chip loading (0.14 mg ml^−1^ final concentration). The drop in pH upon mixing with crystal slurry initiated NO release into solution to bind with the protein.

### 2.3. DtpB sample preparation

DtpB was over-expressed, purified and microcrystals were grown as described previously (Lučić *et al.*, 2020 ▸). In brief, expression took place in *E. coli*, with LB cultures supplemented with 5-amino­levulinic acid and iron citrate and placed under a carbon monoxide environment following induction with iso­propyl β-d-thiogalactopyran­oside. Purified protein was concentrated to 6–10 mg ml^−1^ and mixed in a microcentrifuge tube with an equal volume of precipitant solution containing 150 m*M* HEPES pH 7.5, 150 m*M* MgCl_2_ and 20%(*w*/*v*) PEG 4000. Crystals 8–10 µm long grew after 24–48 h [Fig. S2(*b*)]. As with McCP-β, the NO photocage was dissolved to 10 mg ml^−1^ in a separate mother liquor solution, then mixed with a concentrated crystal slurry and soaked for 10 min.

### 2.4. UV–Vis spectroscopy

UV–Vis spectroscopy was carried out using a Varian Cary 60 spectrophotometer. Protein concentrations of McCP-β and DtpB were determined using extinction coefficients (ε) of ε_400_ = 70 m*M*^−1^ cm^−1^ for McCP-β and ε_280_ = 18575 *M*^−1^ cm^−1^ for DtpB. Samples for both proteins in the ferric heme state were prepared in phosphate-buffered saline (PBS) solution pH 7.4. A 4.6 *M* stock solution of the synthesized NO cage was prepared in PBS pH 7.4 and an aliquot was added to McCP-β and DtpB to give a final NO cage concentration of 23 m*M*. Absorbance spectra were collected before and after brief exposure to white light from a photographic flash. Spectra of the ferric NO-bound forms of McCP-β and DtpB were also obtained by addition of PROLI NONOate.

### 2.5. SSX data collection at Diamond

Serial synchrotron crystallography (SSX) experiments were performed at beamline I24, Diamond Light Source (UK), using a fixed target approach to rapidly raster silicon chips through the X-ray beam (Horrell *et al.*, 2021 ▸; Owen *et al.*, 2017 ▸). Typically, loaded chips were sealed on each side using a 6 µm layer of Mylar film to prevent evaporation and crystal drying prior to and during data collection. For NO cage experiments, different sealing films were evaluated (Section S3) with the result that the layer of Mylar facing the optical and X-ray beams was replaced by 12.5 µm EVAL EVOH EF-F film (Kuraray; https://eval.kuraray.com/en-emea/products/eval-monolayer-film/). EVAL allows high transmission of 308 nm laser light while maintaining a humid crystal environment and minimizing X-ray scatter. SSX experiments at Diamond beamline I24 used an X-ray energy of 12.4 or 12.8 keV and beam size of 20 × 20 µm.

**Figure 1:**
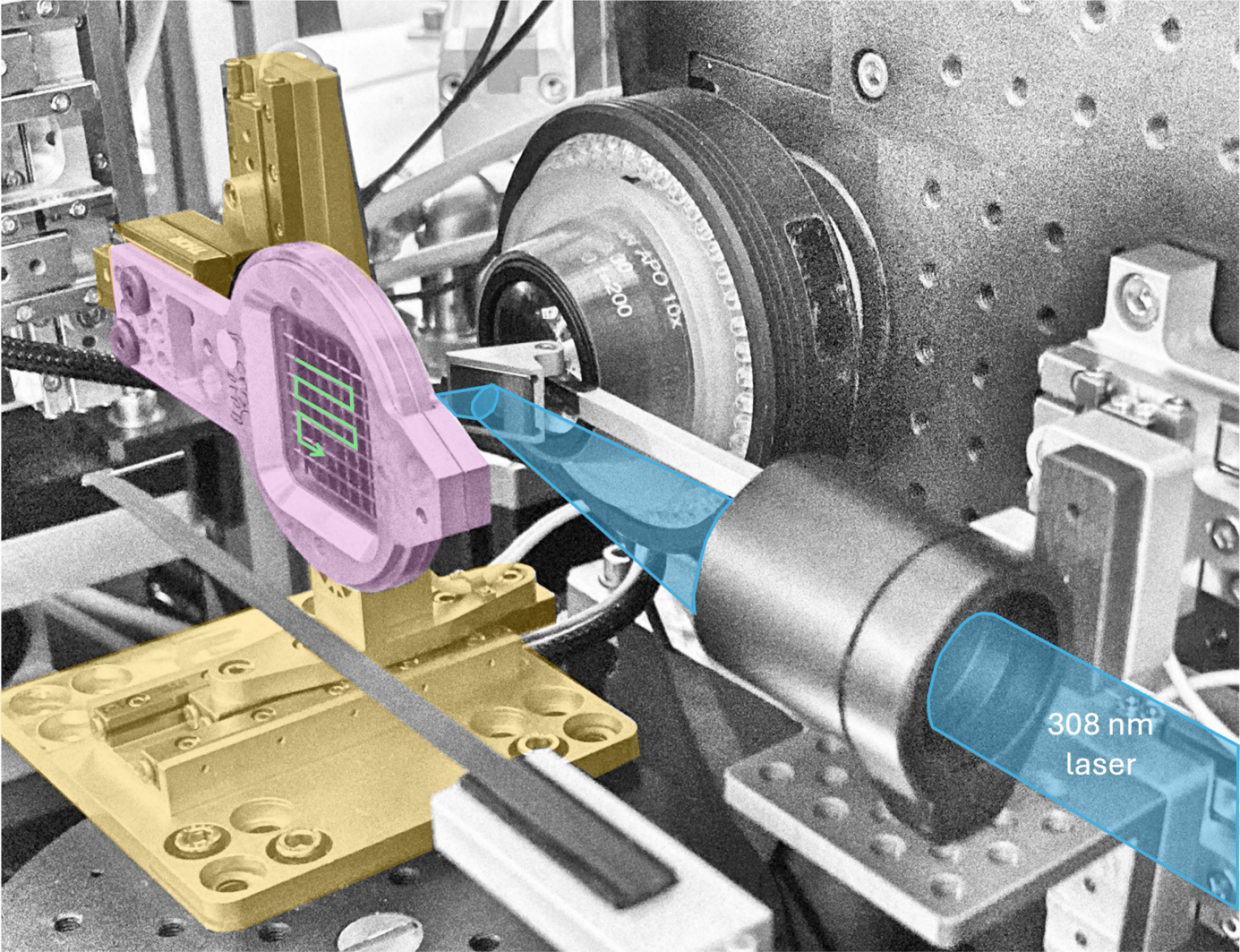
Fixed target motion and laser light delivery hardware in place at Diamond beamline I24, with the chip highlighted in pink, motion stages in gold and laser light path shown in blue. A similar experimental setup was utilized at SACLA, shown in Fig. S5.

Time-resolved experiments at I24 utilized PORTO (PORTable pump-prObe), a femtosecond pulsed laser system consisting of two modules: a diode pumped, solid state laser, based on an Yb:KGW regenerative amplifier, Pharos from Light Conversion (LC); and an Orpheus optical parametric amplifier, OPA, again from LC. The pump laser can deliver a maximum of 20 W average power at 1028 nm and has a variable repetition rate from single pulse up to 600 kHz with each laser pulse duration equal to 300 fs. The output of the OPA covers a wide wavelength range spanning from 210 to 2600 nm. For applications requiring high energy per pulse in the visible and UV regions there are second- and fourth-harmonic generation options available which allow maximum conversion efficiency of the fundamental frequency at these specific wavelengths. The pump laser employs an electro-optic modulator, which is used for contrast enhancement and to control the number of pulses delivered in the output of the amplifier. The laser beam is delivered to the interaction region/sample position by an optical setup combining mirrors and an achromatic focusing lens. The achromatic lens is mounted on a motorized two axis stage (SmarAct) allowing for fine tuning in the vertical and along the beam propagation directions and focuses the light down to a diameter of a roughly 50 µm (FWHM) spot at the sample position. The laser focal spot size at the sample position was measured using knife-edge scans in both the horizontal and the vertical. Laser light was coupled to the sample position by a right-angle prism mirror and was approximately 15° off-axis from the X-ray beam (Fig. 1 ▸).

To control the laser energy reaching the sample position, neutral density (ND) filters (Thorlabs) were inserted into the beam and the repetition rate of the laser was kept constant at 5 kHz (50 kHz in earliest experiments). With no ND filter, the laser imparted a total energy of 19 µJ to the sample area over a period of 5 ms (25 pulses). The laser power at the sample position was attenuated by up to 99% to investigate the dependence of photocage release and the occupancy of the ligand bound states on the laser power. Reaction initiation used an excite and visit again (EAVA) strategy, where the apertures on the fixed targets are visited twice. Laser excitation takes place on the first pass, before returning to collect diffraction data from the same wells after the requested delay time has passed. This approach allows a wide range of timepoints to be collected in a time efficient manner (Schulz *et al.*, 2018 ▸). The timing scheme of fixed target motion, laser initiation and diffraction data collection is shown in Fig. S6(*b*).

### 2.6. SACLA SFX setup

Viscous extruder data for McCP-β were collected at SACLA,^1^ with the extruder installed as part of the *DAPHNIS* (*Diverse Application Platform for Hard X-ray Diffraction in SACLA*) system on BL2 (Tono *et al.*, 2015 ▸). The viscous hy­droxy­ethyl cellulose medium was dissolved to 200 mg ml^−1^ in a 1 mg ml^−1^ solution of photocage in mother liquor under red light. Crystals soaked with photocage, as described above (Section 2.2), were then mixed with the viscous medium. X-ray data were collected at the 30 Hz repetition rate of SACLA with light-initiated and dark structures collected in an alternating fashion by triggering the 308 nm UV pump laser at 15 Hz. Light structures were obtained using a 10 ms pump–probe time delay. Unlike at I24, pump laser illumination utilized a single intense pulse of 5 ns duration, rather than multiple pulses over 5 ms.

Fixed target data collection at SACLA utilized the same silicon fixed target chip motion system used at I24, with a portable three axis stage, on-axis viewing camera and control system (Sherrell *et al.*, 2015 ▸) (shown in Fig. S5). Data were collected at 30 Hz, meaning collection from a full chip took approximately 14 min. Reaction initiation was performed using 308 nm laser light output of a tuneable nanosecond laser system, focused to a spot size of 100 µm with pulse energy up to 200 µJ. As at I24, the laser was slightly off axis with the position of the final mirror upstream of the sample position resulting in a laser beam approximately 15° off-axis from the X-ray beam. For light-initiated structures with short delay times (≤ 20 ms), the 308 nm pump laser was triggered before each XFEL pulse, while the chip was already in position. A timing schematic of the pump–probe laser is shown in Fig. S6(*a*).

### 2.7. Data processing

Diffraction data for the structures from I24 were processed using the *xia2.ssx* pipeline which implements algorithms from *DIALS* (version 3.14.2) (Beilsten-Edmands *et al.*, 2024 ▸). Detector geometry was refined during each beam time using *dials.refine*. Resolution cutoffs were chosen where CC_1/2_ for the outer resolution shell fell below 0.3.

The resting state SFX structure of McCP-β was processed using the *Cheetah*/*CrystFEL* pipeline as described previously (Nakane *et al.*, 2016 ▸). The NO-bound McCP-β and DtpB SFX data were processed using *xia2.ssx* (Winter, 2010 ▸; Beilsten-Edmands *et al.*, 2024 ▸). Standard settings were used for all parameters, except where multiple unit cells were present for DtpB. To separate the different unit cell populations, clustering.central_unit_cellwas set sequentially to each identified unit cell and absolute_length_tolerance was reduced as required to avoid overlap. We note that for the DtpB 30 µJ, 100 µs dataset the absolute_length_tolerance parameter was set to 0.4 even though only one unit cell cluster was present. This was due to a limitation in *xia2.ssx* in terms of number of crystals that can be scaled and merged in one dataset, thus only a subset of data could be used.

Molecular replacement was performed with *MOLREP* (Vagin & Teplyakov, 2010 ▸) within *CCP4i2* (Agirre *et al.*, 2023 ▸). *REFMAC5* (Murshudov *et al.*, 2011 ▸) and *Coot* (Emsley *et al.*, 2010 ▸) were used for alternating rounds of refinement and model building. Omit maps were calculated using *Phenix* (Adams *et al.*, 2010 ▸). Validation was performed with *MolProbity *(Williams *et al.*, 2018 ▸) and the *JCSG Quality Control Check* (version 3.1; Elsliger *et al.*, 2010 ▸). Structural figures were generated with *PyMOL* (Schrödinger).

### 2.8. Occupancy estimation

While recent significant progress has been made in estimating the occupancy of intermediate states in time-resolved crystallography (De Zitter *et al.*, 2022 ▸; Barends *et al.*, 2024 ▸), reliable estimation of NO occupancy in the binding pocket of DtpB and McCP-β proved to be a challenge, with standard tools providing differing results. We utilized two approaches for estimating occupancy, the first based on the *B*-factor analysis and the second comparing *F*_o_ − *F*_c_ difference density. These are described below. A third approach for on-the-fly estimation during beam time involved quantifying the height of any positive peak at the expected position of NO in an *F*_o_ − *F*_c_ difference map, following molecular replacement using an apo model. Occupancy was then estimated by comparing the peak height from the NO to the peak height resulting from a similarly sized atom pair (Nδ^1^=Cɛ^1^ atom pair in the nearby proximal histidine residue) also omitted from the molecular replacement model.

The first approach used for occupancy estimation compared *B* factors of atoms in proximity to the NO. The *B* factor of the heme iron remains consistent across modelled NO occupancy values, but systematic variation in the *B* factor of the NO nitro­gen is evident, with lower occupancy values having lower *B* factors, representing a more ordered ligand and a higher electron density peak height. We refined structures with NO modelled in the *F*_o_ − *F*_c_ map features, which were where they were expected in the distal heme pocket, and the occupancy value where the *B* factor of the nitro­gen closest matches that of the iron was taken as the true occupancy of the NO in the experimental data. To determine the *B* factor of NO at all occupancies, *REFMAC* refinement was run in an automated manner with the occupancy varying from 0.00 to 1.00 in increments of 0.01. This allowed the occupancy to be determined as the point where the magnitude of the difference in the *B* factor was minimized. This is demonstrated in Fig. S10, which shows variation in DtpB *B* factors for the highest laser power dataset. This was performed on a per-chain basis, producing six occupancy values for each hexameric DtpB structure, and two for dimeric McCP-β.

Subsequent to our investigation and development of this and other approaches to reliably estimate occupancy, Barends *et al.* (2024 ▸) described investigation of several approaches as part of a study into the effect of laser power on the photodissociation of CO from myoglobin. These included extrapolation of structure factors for full occupancy of the photo-dissociated state (De Zitter *et al.*, 2022 ▸), which was found to systematically underestimate occupancy of simulated data. A multicopy approach was also used, which quantified *F*_o_ − *F*_c_ density where the triggered copy of myoglobin was refined with CO in the dissociated position, while the dark state with bound CO was left unaltered. For our second estimate of occupancy, we used this approach, quantifying the residual *F*_o_ − *F*_c_ difference density peak at the NO position. We took the generated structures with NO modelled at different occupancies in the range 0.00–1.00 and extracted the height of the *F*_o_ − *F*_c_ map at the NO positions after refinement. As with the *B*-factor approach, the structure with the lowest magnitude of the difference density was taken as having the true occupancy.

## 3. Results

### 3.1. UV–Vis characterization

**Figure 2:**
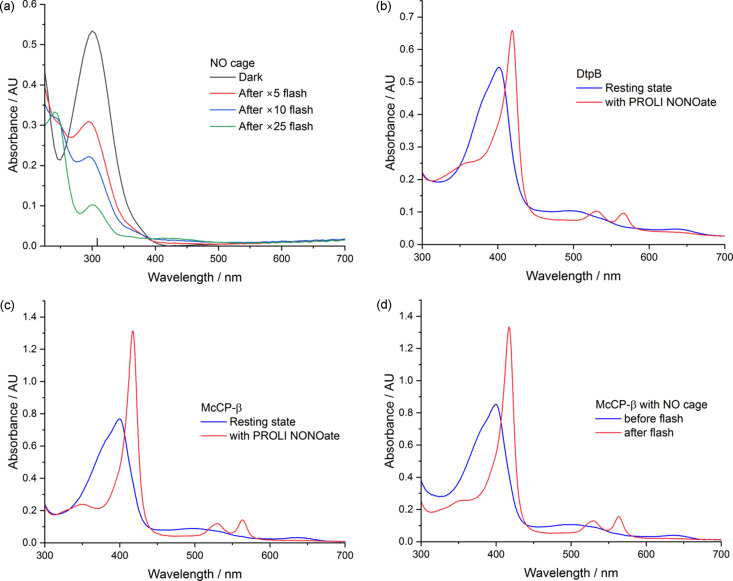
UV–Vis absorbance spectra of the NO cage, DtpB and McCP-β. (*a*) Spectra of synthesized NO cage both before and after exposure to white light from a photographic flash. The *x* axis is marked at 308 nm. (*b*) Spectra of ferric resting state DtpB (blue) and DtpB after addition of PROLI NONOate (red). (*c*) Spectra of ferric resting state McCP-β (blue) and also after addition of PROLI NONOate (red). (*d*) Spectra of ferric resting state McCP-β with NO cage before and after a single white light flash (blue and red, respectively).

UV–Vis absorbance spectra from the synthesized cage were collected as described in Section 2.4 and are shown in Fig. 2 ▸(*a*). As previously reported by Namiki *et al.* (1997 ▸), λ_max_ = 300 nm and, after brief exposure to white light, several spectral changes occur consistent with photolysis and release of NO. Fig. 2 ▸(*b*) shows absorption spectra of DtpB in solution; data for the ferric resting state (blue) and after the addition of 10 µl PROLI NONOate (red) are shown. Upon NO binding, the Soret peak shifts from 401 to 419 nm, and α and β peaks appear in the *Q*-band region (500–750 nm) characteristic of six-coordinate iron heme, *i.e.* with both His and NO as axial ligands. Similar results were obtained for ferric McCP-β and after NO binding following release from PROLI NONOate [Fig. 2 ▸(*c*)]. Upon NO binding the Soret band is shifted from 400 to 417 nm with split *Q* band features at 530 and 563 nm appearing consistent with a ferric NO state of McCP-β. Fig. 2 ▸(*d*) shows the absorbance spectrum of McCP-β in the presence of the NO cage (blue line), with features at the same wavelengths seen in the ferric state in Fig. 2 ▸(*c*) (*i.e.* Soret peak at 400 nm, *Q* band at 503 nm and charge transfer band at 635 nm). We note that the presence of the NO cage does not in any way interfere with the McCP-β spectrum. After a single photographic flash, the absorbance spectrum [red line, Fig. 2 ▸(*d*)] changes to be consistent with that obtained from McCP-β in the presence of PROLI NONOate (*i.e.* Soret band at 417 nm with split *Q* band features at 530 and 563 nm). Spectra were also collected with DtpB in the presence of the NO cage: prior to a flash the absorbance spectrum was identical to the resting state [Fig. 2 ▸(*b*)]. Both in the presence and in the absence of the cage DtpB suffered from photobleaching and we were therefore unable to obtain an NO-bound spectrum by these means.

### 3.2. Resting state, ‘dark’ control and positive control PROLI NONOate structures

**Figure 3:**
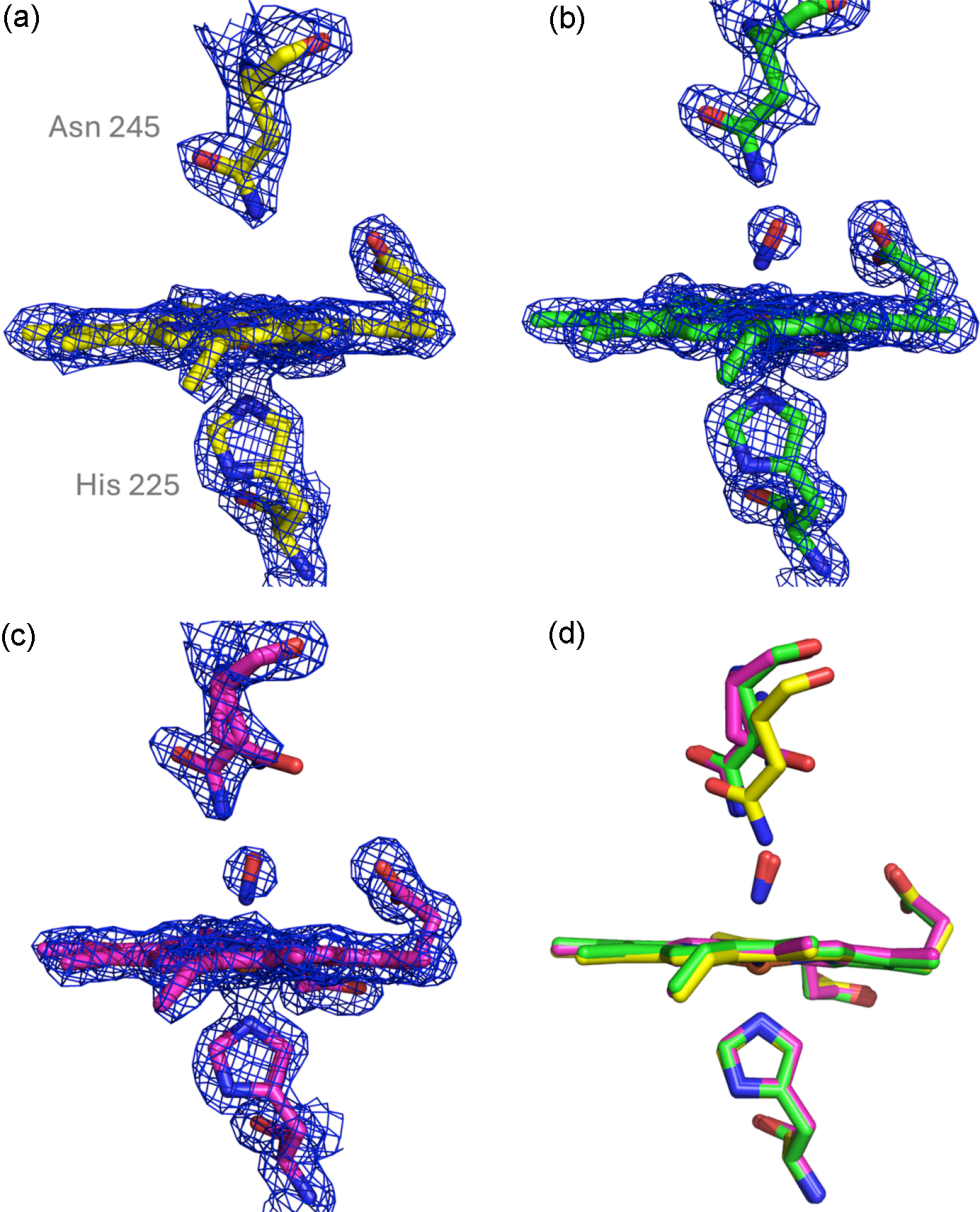
Heme site structures of DtpB. (*a*) Dry heme site in the SFX ferric resting state structure devoid of any distal pocket axial ligand. (*b*) NO-bound heme site obtained at SACLA using the NO photocage 100 µs after 30 µJ laser illumination. (*c*) Heme site from the 100 µs 100 µJ structure showing the multiple conformations of Asn245 evident in some higher occupancy NO-bound states. (*d*) Overlay of structures shown in (*a*) yellow, dry heme site; (*b*) green, NO-bound 100 µs, 30 µJ; and (*c*) pink, NO-bound 100 µs, 100 µJ. Note a small shift in main-chain position for Asn245 in the dry heme site structure. In all panels chain A is shown.

Prior to utilizing the NO cage to characterize NO binding, we sought to obtain suitable control structures including both the ground state of the proteins (ligand free) and a ‘dark’ structure where the NO cage compound had been soaked into crystals but not activated by the laser pulse. The SFX resting state structure of ferric DtpB has been previously described (Lučić *et al.*, 2020 ▸), revealing a five-coordinate heme with a ‘dry’ distal heme pocket devoid of water molecules [Fig. 3 ▸(*a*)]. The SSX resting state structure (2.75 Å resolution) of DtpB shows no significant differences compared with the SFX structure (Fig. S9).

For comparative purposes we show here high-occupancy structures generated using the photocage system [Figs. 3 ▸(*b*) and 3 ▸(*c*), 1.52 and 1.67 Å resolution], described in further detail in Section 3.3. On binding to the heme, NO was observed to bind in an end-on fashion via the nitro­gen atom with an Fe—N distance of approximately 2.0–2.5 Å and an Fe—N—O angle of approximately 105–150°. The variability in these values occurs across the six independent heme groups within the crystallographic model and could represent either genuine differences in binding due to subtle differences in the crystalline environments or simply uncertainty in modelling a small ligand into the electron density features. Interestingly, our previous structure of DtpB where an Fe(IV) state had been produced by soaking of the crystals with hydrogen peroxide also showed variability in the Fe—O bond length between the different monomers of the hexamer (Lučić *et al.*, 2020 ▸). The structure of the heme pocket was only mildly altered by binding of NO [Fig. 3 ▸(*d*)]. A small shift in the side-chain position of Asn245 was observed compared with the ‘empty’ site structure in all six of the hemes, and at all laser powers and timepoints. In the higher occupancy heme site structures, a double conformation of Asn245 was observed in chains A, E and F, with the amide group in the additional conformation pointing away from the heme [Fig. 3 ▸(*c*)]. In all NO-bound structures the oxygen atom of the NO was positioned to form a hydrogen bond with either Asn245 or Arg243 (see Figs. 3 ▸ and 6 for side-chain positions). The proximity of the O atom in these NO-bound states is of interest in relation to the previously proposed peroxidase mechanism in DtpB, where Arg243 has been implemented to act as proton acceptor and donor to facilitate the heterolytic cleavage of the O—O bond in compound 0 [Fe(III)—O—OH] which is generated following binding of H_2_O_2_ to the Fe(III) heme prior to formation of the long-lived compound I (Lučić *et al.*, 2020 ▸).

**Figure 4:**
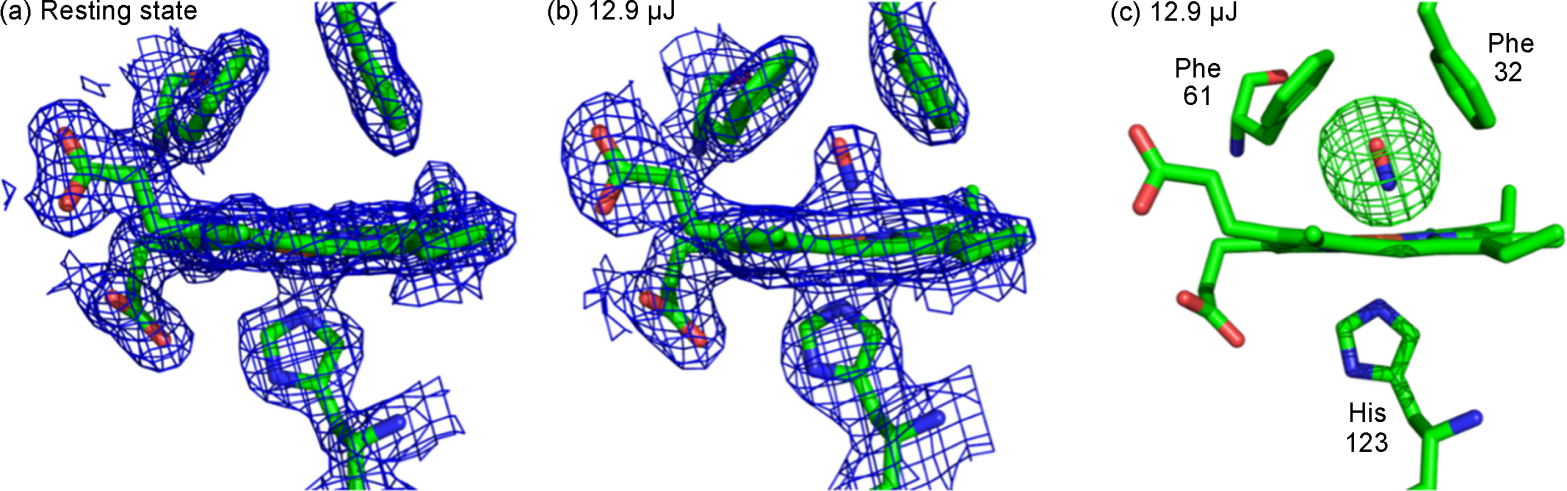
SFX structures of McCP-β, showing chain A. 2*F*_o_ − **F*_c_* maps for (*a*) a resting state heme site collected from a fixed target chip and (*b*) an NO-bound heme site 20 ms after using the NO cage in a viscous extruder, both contoured at 1σ. The NO occupancy is 0.7. (*c*) Omit map contoured at 5σ for the NO-bound structure.

In a similar manner to DtpB, the resting state ferric SFX structure of McCP-β was determined at SACLA to 1.80 Å resolution [Fig. 4 ▸(*a*) and Table S1 of the supporting information]. This represents the first room-temperature structure of McCP-β, and a detailed comparison with the previously determined 100 K structure is given in Section S5. The only significant difference observed is displacement of Phe32, which guards the distal heme pocket from the adjacent solvent channel, away from the heme group. We also utilized the pH-dependent NO donor PROLI NONOate to obtain a highly occupied NO-bound structure of McCP-β to 1.85 Å resolution. Data collection and processing statistics for these structures are shown in Table S1. In the NO-bound structure generated with PROLI NONOate, clear electron density in difference maps indicates NO binding, and allows modelling of an NO molecule at full occupancy, in a bent geometry with Fe—N bond distances of 1.91 and 1.90 Å, and Fe—N—O angles of 134 and 153°, in chains A and B, respectively [Figs. S7(*c*) and S7(*d*)]. The highly hydro­phobic and water-free heme site leads to very few structural changes between the apo and NO-bound forms. The Phe32 residue highlighted above exhibits a small further displacement from the heme in the NO-bound structures, providing space for the NO [Figs. S7(*e*) and S7(*f*)]. This room-temperature NO-bound structure is compared with an equivalent structure collected at 100 K (Adams *et al.*, 2023 ▸) in Figs. S7(*a*) and S7(*b*). The comparison reveals minimal structural differences due to temperature, although the cryogenic structure exhibits multiple conformations of the NO in chain B. When the NO cage is soaked into the crystals, but no laser is used, NO is absent in the distal heme pocket of McCP-β [Fig. 4 ▸(*a*), 2.20 Å resolution], confirming the cage does not ‘leak’ and that loading, transfer and alignment protocols do not result in NO release. No evidence of the photocage molecule is seen bound within the crystal, instead remaining in the disordered bulk solvent.

### 3.3. Laser initiated NO binding

**Figure 5:**
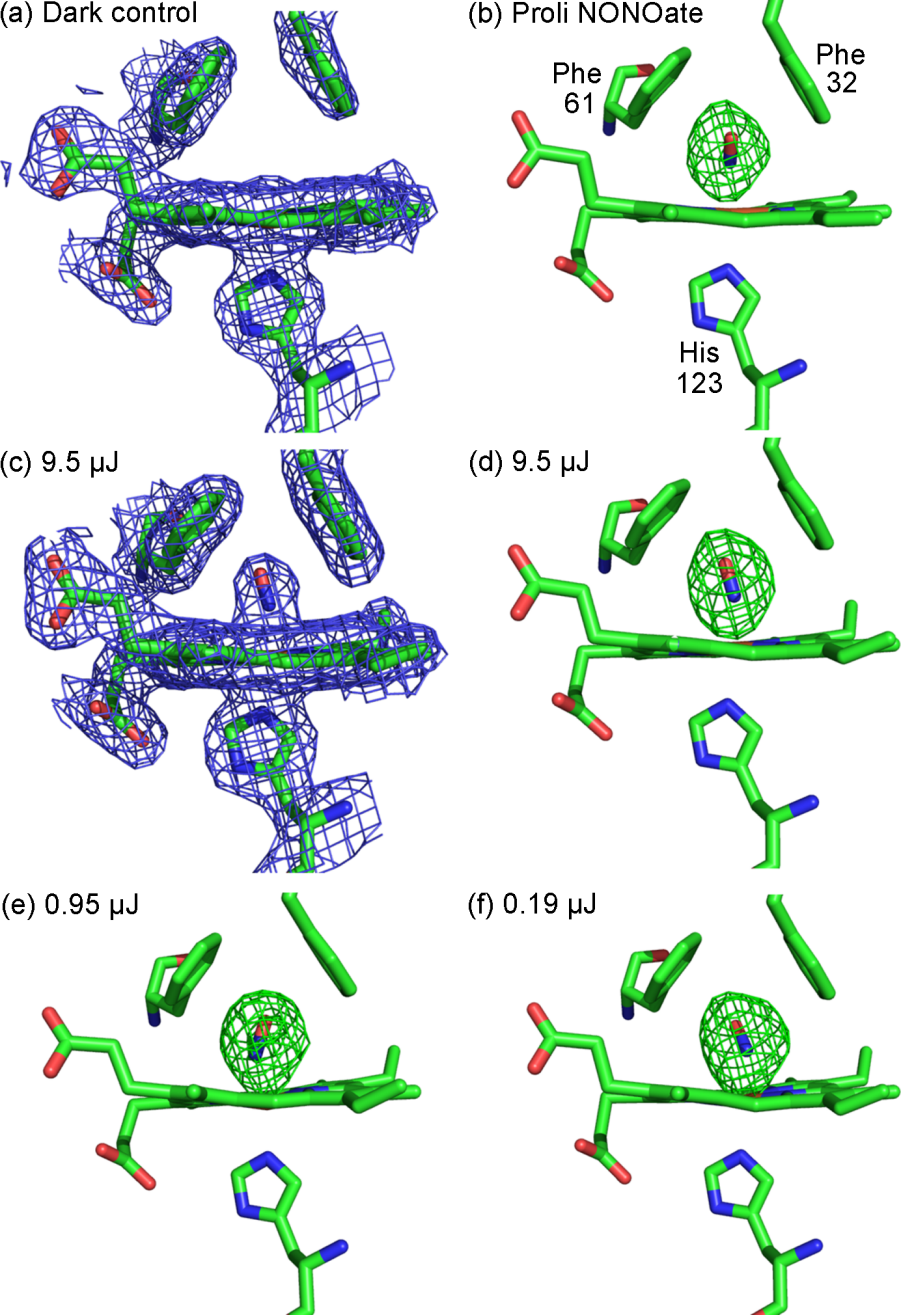
Comparison of the heme site in chain A of McCP-β SSX structures. 2*F*_o_ − **F*_c_* maps of (*a*) the dark control structure and (*c*) the laser illuminated NO cage structure, both contoured at 1σ. (*b*) and (*d*)–(*f*) Omit maps contoured at 5σ for McCP-β SSX structures, either (*b*) soaked with PROLI NONOate, or from time-resolved datasets 1.44 s after NO release from the photocage, collected with laser powers of (*d*) 9.5 µJ, (*e*) 0.95 µJ and (*f*) 0.19 µJ. The NO occupancies in (*b*)–(*f*) are all 1.0.

The observation of NO bound to McCP-β in experiments using PROLI NONOate as a donor allowed us to proceed to photocage experiments. The first laser-initiated data collections used a delay time (1.44 s) much longer than the predicted NO release (microseconds; Section 2.1) and binding times [<1 ms; (Adams *et al.*, 2023 ▸)], for simple verification of NO release from our cage and successful binding to form a stable end-state complex. To establish suitable parameters for effective release of the photocage without the use of excessive laser power that could cause sample heating or multi-photon excitation effects, we conducted a laser power titration using the same fixed time delay of 1.44 s between laser and X-rays. These structures were determined from data collected after optimization of the experimental setup which allowed much lower laser powers to be used. Three SSX datasets were collected at different laser powers under otherwise identical conditions, at 9.5, 0.95 and 0.19 µJ (approximately 5, 0.5, 0.1 nJ µm^−2^), to resolutions of 2.00, 1.80 and 1.75 Å, respectively. These datasets yield similar protein structures, with full NO occupancy displayed in all of them, as shown in Fig. 5 ▸(*c*)–5(*f*). The binding of NO in the different structures exhibits a similar Fe—N bond length, and some variation in the Fe—N—O bond angle, but the direction in which the bent NO is oriented is not correlated with laser power. Instead, this is relatively poorly determined due to the difficulty of fitting the small diatomic ligand into the electron density.

**Figure 6:**
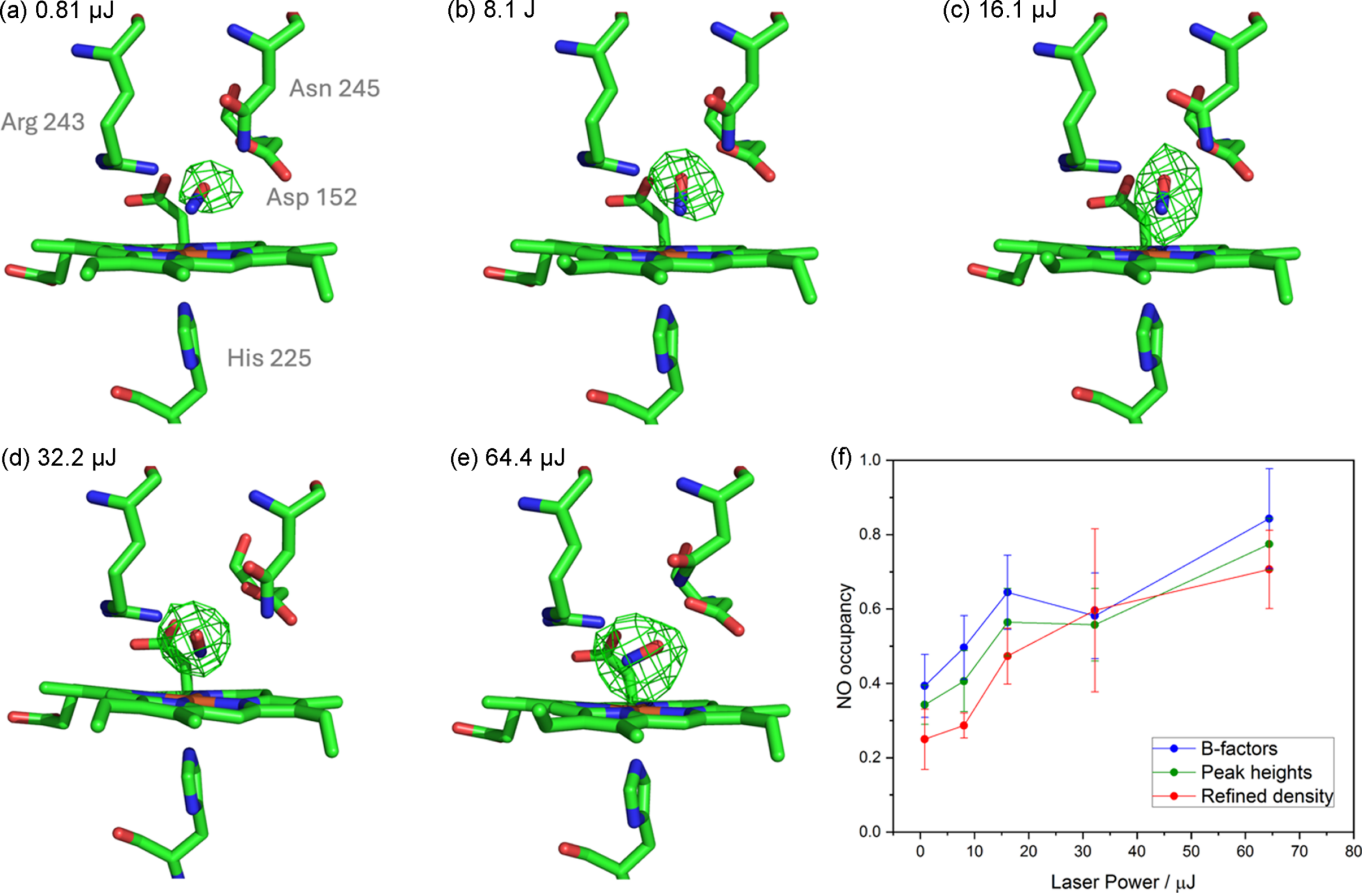
Laser-activated SSX structures of DtpB. (*a*)–(*e*) Omit electron density, contoured at 5σ, maps for NO-bound heme site in chain C of DtpB, collected with different laser energies of 0.81, 8.1, 16.1, 32.2, 64.4 µJ (approximately 0.4, 4, 8, 16, 32 nJ µm^−2^). With increasing laser power, the electron density maps support the presence of an NO molecule bound to the heme iron with increasing partial occupancy. The occupancies for the different laser powers were 0.81 µJ = 0.39, 8.1 µJ = 0.50, 16.1 µJ = 0.65, 32.2 µJ = 0.58, 64.4 µJ = 0.84 and are summarized in Table S4. (*f*) Occupancy of NO for DtpB SSX structures collected with different laser energies, averaged across all six chains, as determined by three methods shown in blue (*B*-factor comparison), green (*F*_o_ − *F*_c_ map peak height) and red (multicopy refinement). Error bars show one standard deviation in the mean. Occupancy values in the deposited structures are taken from the occupancy refinement. Scaling and refinement statistics can be found in Table S2.

A series of five NO-bound SSX structures of DtpB solved to 2.40 Å resolution were also collected in the same manner, using different laser powers (Fig. 6 ▸). This power titration shows a rapid increase and then a levelling off in NO occupancy as a function of laser power [Fig. 6 ▸(*f*)], but no correlation of Fe—N bond length or Fe—N—O bond angle with laser power. Similarly to the McCP-β SSX structures, the orientation of the bent NO is poorly determined, partly due to the lower resolution of these structures

It is instructive to consider our initial fixed target SSX photocage experiments where we used significantly higher laser powers and variable pulse rates to be able to successfully initiate and observe *in crystallo* photolysis of the NO cage. An initial high power laser experiment delivering 1600 µJ (580 nJ µm^−2^) of laser energy to the sample over 10 ms of 50 kHz PORTO laser pulses showed NO binding with an occupancy of 1.0 (Fig. S8, 1.80 Å resolution) but with notably different 2*F*_o_ − *F*_c_ and omit density features to those from subsequent data collections. This high laser power requirement was due, at least in part, to an initial sub-optimal experimental setup where the fixed targets were orientated with the narrow end of the funnel-like apertures facing the laser beam, and with Mylar sealing both sides. In this orientation, the chip acts as an aperture, blocking a significant fraction of laser light resulting in inefficient excitation and causing only part of the crystal volume seen by X-rays to be illuminated by the laser. The unexpected electron density features may therefore be due to laser induced damage (though a loss in diffracting power was not observed) or non-uniform excitation. All subsequent data collections, and all data presented here, utilized chips orientated with the wide end of the aperture facing the laser beam and EVAL film sealing the laser-facing side of the chip. Further, we found results to be most reproducible when the total laser power incident on the sample was varied using ND filters rather than by changing the pulse rate.

### 3.4. Time-resolved SFX using the NO cage

After collecting NO-bound structures at long timepoints using synchrotron radiation, confirming *in crystallo* excitation of the NO cage led to binding in the protein, we sought to collect data at shorter timepoints using SFX to show the rapid nature of reaction initiation from the photocage. Due to travel restrictions caused by the COVID-19 pandemic, our first time-resolved SFX experiments at SACLA used a viscous extruder to deliver microcrystals of McCP-β, prior to photoexcitation, as previously described for time-resolved studies of NO binding to cytochrome P-450 NOR (Tosha *et al.*, 2017 ▸). With a 20 ms delay time and laser power of 12.9 µJ (∼3 nJ µm^−2^), we determined an McCP-β structure to 2.0 Å resolution. This structure exhibits NO bound to Fe at 0.7 occupancy in both chains, revealing that photocage photolysis and NO binding are complete within 20 ms [Figs. 4 ▸(*b*) and 4 ▸(*c*)]. This represents a useful control given that the only previous room-temperature SFX structure using the NO photocage had been carried out with a high viscosity extruder sample delivery system (Tosha *et al.*, 2017 ▸). The lower occupancy in this structure compared with the SSX structures of McCP-β is likely due to laser light being scattered by the viscous hydroxy­ethyl cellulose medium in which the crystals are embedded. This laser power is approximately three times lower than the 30–50 µJ used for previous experiments with the same cage and viscous medium (Tosha *et al.*, 2017 ▸), which also achieved NO occupancy of 0.7. Aside from the NO occupancy, the structure shows no significant differences to the SSX structures or the resting state SFX structure, indicating the environment in the viscous medium versus that in the mother liquor has little effect on the protein crystals.

**Figure 7:**
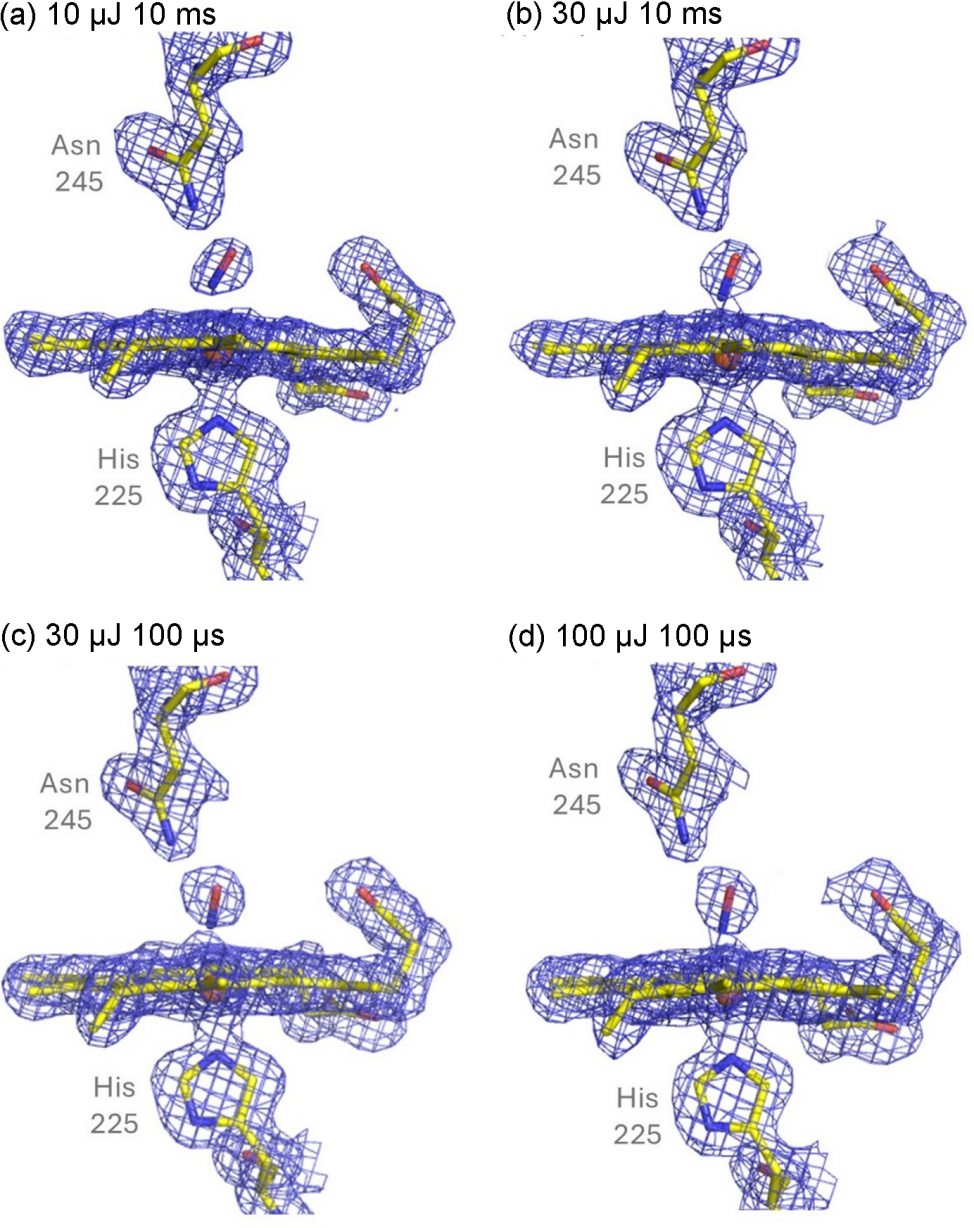
2*F*_o_ − **F*_c_* electron density maps contoured at 1σ for a representative heme group (chain C) of DtpB with different laser powers and time delay parameters obtained using fixed target SFX at SACLA. Clear electron density for the bound NO molecule is evident at 10 ms using 10 µJ laser power with an occupancy of 0.35 (*a*). An increase of laser power to 30 µJ increases the occupancy of NO to 0.45 (*b*). A faster timepoint at 100 µs at 30 µJ has a slightly lower occupancy (0.30), suggesting a time dependence of binding (*c*). At this faster timepoint, a further increase of laser power to 100 µJ again increased the occupancy to 0.45 (*d*).

In subsequent time-resolved SFX experiments using the same fixed target sample environment as the SSX experiments, we collected DtpB structures with time delays of 100 µs and 10 ms with incident laser power between 10 and 200 µJ (∼2–40  nJ µm^−2^) (Fig. 7 ▸) solved to resolutions of 1.69–1.52 Å. Scaling statistics for these data are given in Table S3. The highest available laser power (200 µJ) resulted in severe damage to crystals from the laser with a greatly reduced number of indexed diffraction patterns obtained per chip under identical loading regimes (data not shown). At lower laser powers, difference density supporting the binding of NO is evident within the first 100 µs [Figs. 7 ▸(*c*)–7 ▸(*d*)]. The occupancy for the refined NO at this timepoint increased from 0.30 to 0.45 when the laser power was increased from 30 to 100 µJ (∼6–20 nJ µm^−2^). These data indicate that release of the photocage is effective even at the lowest laser power used, and sufficient photoproduct can diffuse throughout the crystal on this short timescale to allow structure determination of the bound form with reasonable occupancy. With a longer 10 ms timepoint, the occupancy is slightly higher at each power level but is again power dependent [Figs. 7 ▸(*a*) and 7 ▸(*b*)]. Comparison of these different structures revealed no significant changes in the binding geometry of NO or nearby residues in the heme pocket, meaning that only a two-state transition between unbound and bound states is observed. This suggests that any intermediate steps in the NO binding process must be complete on timescales of less than 100 µs.

## 4. Discussion

We have demonstrated a sample and time efficient approach using fixed targets to carry out time-resolved SSX and SFX experiments with an NO photocage over the microsecond to second time regimes. Approximately 4 mg of protein was required per combination of timepoint and laser power. Using the same fixed target sample delivery methodology at XFEL and synchrotron beamlines over all desired timepoints enables the results of experiments conducted at these different facilities to be directly compared without confounding experimental factors. Effective photocage release occurred for both protein samples in a laser-power-dependent manner with suitable photoactivation parameters established using longer timepoints in a time-resolved experiment. This enabled subsequent experiments to access faster time regimes with NO binding to DtpB occurring even at the fastest 100 µs timepoint. This may be compared with the photochemistry previously characterized for the NO cage where a fast laser pulse releases NO on the microsecond timescale (Namiki *et al.*, 1997 ▸). It is evident that at least for the protein systems used in this work, photocaged gas ligands can effectively enable time-resolved crystallography on sub-millisecond timescales. Currently, such time regime experiments are largely restricted to XFELs or the very brightest synchrotron beamlines, but new, faster detector and beamline technology will enable the microsecond time regime to be accessed more routinely in coming years (Orlans *et al.*, 2025 ▸).

Assessment of occupancies in light-activated processes remains challenging, particularly for small ligands such as the diatomic NO. For our experiments with DtpB, we found both *B*-factor and *F*_o_ − **F*_c_* peak height quantification to give results that display the same occupancy trend with laser power, but both generally overestimate NO occupancy compared with multicopy refinement [Fig. 6 ▸(*f*) and Table S4]. This may be due to NO having higher mobility in the distal heme pocket compared with the iron atom, resulting in lower *B* factors for NO than Fe even if the occupancies are the same. The on-the-fly approach was found to report occupancy values within 0.10 of the occupancy determined using the other approaches and provided a useful guide to the success of a particular experiment that could be obtained during the beam time itself, enabling for modification of experimental conditions where necessary.

An intriguing observation was that of differing occupancies for the released NO within the different (chemically identical) monomers of the homo-oligomeric proteins, particularly for the hexameric DtpB (in contrast, McCP-β is a homodimer). An advantage of the photocage method is that faster timepoints may be accessed because the photocage is pre-soaked into the crystals and so diffusion is less of an issue. Nonetheless, occupancies in different monomers were rather different. One possible explanation would be differences in the proximity to the bulk solvent channels in which the photocage molecules presumably are present, although no correlation was seen between chains with higher NO occupancy between the differing laser powers. Differences may also be a consequence of uncertainty in NO occupancy determination. Our work therefore highlights the importance of the crystalline lattice and solvent channels within it in enabling proximity of the NO cage to each active site and ability of the released ligand molecules such as NO to migrate to these via diffusion. The concentration of photocage within the crystal is relatively modest (< 20 m*M*), in McCP-β this concentration corresponds to approximately 15 photocage molecules, or 21 NO molecules assuming a quantum yield of 1.4, per unit cell. For a 30 µm McCP-β crystal soaked with NO cage at 10 mg ml^−1^, transmission of the UV laser is ∼16%. This equates to absorption of approximately 16 photons per unit cell at the lowest laser power used (0.19 µJ), which is within the single photon regime. Given McCP-β contains 24 heme groups per unit cell and high occupancy bound NO is observed, cage release and the subsequent binding of photocage-released NO is an efficient process. We speculate it may be possible to use lower laser powers if the cage concentration is increased. These considerations may factor into the selection of particular crystal forms for time-resolved SSX or SFX using soaked-in photocages and implies that generalized rules for photocage concentration and laser parameters may not apply, and these should in fact be determined for new systems using on-the-fly estimates of occupancy of released molecules or the resulting intermediate states.

The level of NO occupancy and its relation to laser power were different for the two ferric proteins. Ferric heme proteins have lower affinity for NO than ferrous heme (Cooper, 1999 ▸), and it is possible that the observed occupancy differences could be related to differences in ferric heme NO affinity. However, other differences between the microcrystalline proteins could also influence the observed occupancy such as the diffusion of cage and released NO through the solvent channels, differences in the crystalline mother liquor within those channels and slightly different crystal morphologies.

Much attention has been paid recently in ensuring that the biological interpretations of laser activated studies of naturally light-activated proteins are not compromised by multiphoton effects (Grünbein *et al.*, 2020 ▸; Barends *et al.*, 2024 ▸). Notably for the direct photodissociation of CO from myoglobin, multiphoton events led to artefactual structural changes to the protein, while careful attention to ensuring single photon conditions in contrast allowed the true dynamics of CO dissociation to be characterized. In the case of photocage experiments, it might be expected that the inherent activity of the protein itself is not directly affected by the laser pulse like a naturally light-activated protein, and so multiphoton effects are much less of a concern (contributing to the overall release of the caged compound). The goal is instead to maximize the occupancy of the bound ligand at the protein active site to aid in structure interpretation. In the case of DtpB, we observed lattice destruction in the form of a greatly reduced hit-rate at the highest laser power (200 µJ), but no other systematic structural changes, beyond variation in NO occupancy, occur as a function of laser power in the lower power region (0.8–100 µJ). This was also true in the low power region (0.2–13 µJ) covered in the McCP-β experiments. This absence of power dependence may reflect a lack of laser-induced damage or be due, in part, to the relatively slow (>100 µs) time regimes probed. The energy deposited by the PORTO laser being spread over 25 pulses at 5 kHz, rather than occurring in a single intense pulse, allows dissipation of the energy between pulses and this may also contribute to an observed absence of laser-induced damage.

While primarily focused on developing a widely applicable photocage system, our results provide insights into the binding of NO to the two different target proteins. In McCP-β, we were able to show that the photocage successfully generated the initial distal six-coordinate species that is an end state in this protein but a transient intermediate in other cytochromes *c*′ (Hough & Andrew, 2015 ▸). We can therefore anticipate that time-resolved, photocage initiated SFX will be able to capture the transient intermediates in future studies. For DtpB, the structure of the NO ferric heme complex of this or any other B-type DyP have not been previously determined, and thus we have demonstrated here that such a complex rapidly forms on exposure to NO after photocage release. As noted previously, the function of DtpB in *S. lividans* is unknown, but B-type DyPs are always located in the bacterial cytoplasm, often the site of NO production. Furthermore, B-type DyPs are generally considered to be poor peroxidases (Lučić *et al.*, 2022 ▸; Lučić *et al.*, 2024 ▸). Considering the present study, it could be that DtpB belongs to a growing body of proteins known as moonlighting proteins that have multifunctionalities (Werelusz *et al.*, 2024 ▸). This work therefore provides scope for future studies to investigate the potential role of NO binding to DtpB in *Streptomyces lividans* and determine whether it has a role in NO signalling pathways.

We have described experiments using an NO photocage using both synchrotron and XFEL radiation utilizing a high-viscosity extruder and fixed targets. The dependence on laser power of the observed NO density at the active sites of two proteins was demonstrated, revealing that, in our systems, increasing the power deposited in the crystal increases the quantity of NO and hence ligand occupancy. Our work demonstrated that low laser powers (0.19 µJ, ∼0.1 nJ µm^−2^) resulted in effective photocage release. Multiple timepoints were explored given that the microsecond timescale for release of NO from the photocage is followed by a diffusion and binding process which may be considerably slower, especially given the widely reported differences in reaction rates of proteins and enzymes in crystals compared with in solution (Efremov *et al.*, 2006 ▸; Konold *et al.*, 2020 ▸; Aumonier *et al.*, 2022 ▸). We note that this work cannot rule out the possibility that NO released from the photocage within crystals in a particular aperture of the fixed target could diffuse into adjacent apertures as a gas. Removal of excess solution from around the crystals during loading means that there should be no through-liquid pathway between apertures, but high concentrations of NO generated within the crystal could in principle move into the headspace between the aperture and the sealing film.

Our work has focused on establishing suitable conditions for the effective use of the NO cage using the sample efficient and flexible fixed target approach. This sample delivery method enables a wide range of timescales to be accessed under identical experimental conditions. This in turn will enable the ability to obtain stop-motion movies of enzymatic reactions, or other protein processes such as signalling that are initiated by NO over a very wide range of timescales from microseconds to seconds. Combining this methodology with *in situ* spectroscopic data obtained from crystals will add further capability to track reactions after photocage release.

## 5. Related literature

The following references are cited in the supporting information: Keedy *et al.*(2014 ▸); Kekilli *et al.* (2017 ▸).

## Supplementary Material

Supporting figures and tables. DOI: 10.1107/S2052252525006645/car5004sup1.pdf

PDB reference: DtpB SSX resting state, 9i4q

PDB reference: DtpB SSX photocaged NO 0.8µJ, 9iaa

PDB reference: DtpB SSX photocaged NO 8µJ, 9ia9

PDB reference: DtpB SSX photocaged NO 16µJ, 9i6g

PDB reference: DtpB SSX photocaged NO 32µJ, 9i4u

PDB reference: DtpB SSX photocaged NO 64µJ, 9i4s

PDB reference: DtpB SFX photocaged NO 10µJ 10ms, 9hl1

PDB reference: DtpB SFX photocaged NO 30µJ 100µs, 9hxx

PDB reference: DtpB SFX photocaged NO 30µJ 10ms, 9ho7

PDB reference: DtpB SFX photocaged NO 100µJ 100µs, 9hyv

PDB reference: McCP resting SFX, 9hqt

PDB reference: McCP resting SSX, 9hu1

PDB reference: McCP dark control, 9hyz

PDB reference: McCP ProliNO, 9hs8

PDB reference: McCP photocaged NO 0.19µJ, 9htt

PDB reference: McCP photocaged NO 0.95µJ, 9htv

PDB reference: McCP photocaged NO 9.6µJ, 9q86

PDB reference: McCP photocaged NO 12.9µJ, 9qme

PDB reference: McCP photocaged NO 1600µJ, 9htc

## Footnotes

1 Due to the COVID-19 travel restrictions between the UK and Japan, extruder experiments were conducted remotely with the assistance of SACLA beamline staff.
