# Supplementary material for: Time-resolved serial synchrotron and serial femtosecond crystallography of heme proteins using photocaged nitric oxide
Source: IUCrJ. 2025 Aug 22;12(Pt 5):582–94. doi: 10.1107/S2052252525006645 (PMC12403166; doi:10.1107/S2052252525006645)
Supplement: Supplementary file 1 [file m-12-00582-sup1.pdf]

IUCrJ

Volume 12 (2025)

Supporting information for article:

Time-resolved serial synchrotron and serial femtosecond crystallography of heme proteins using photocaged nitric oxide

Peter Smyth, Sofia Jaho, Lewis J. Williams, Gabriel Karras, Ann Fitzpatrick, Amy J. Thompson, Sinan Battah, Danny Axford, Sam Horrell, Marina Lučić, Kotone Ishihara, Machika Kataoka, Hiroaki Matsuura, Kanji Shimba, Kensuke Tono, Takehiko Tosha, Hiroshi Sugimoto, Shigeki Owada, Michael A. Hough, Jonathan A.R. Worrall and Robin L. Owen

S1. Synthesis of nitric oxide photocage

Synthesis of *N,N′* -Bis-(carboxymethyl)-*N,N′* -dinitroso-1, 4-phenylenediamine (compound 4, figure S1) - NO cage - was carried out based on a procedure previously described by (Cabail *et al.*, 2002) in two steps with some modifications. *N,N′* -Diacetyl-1,4-phenylenediamine (98 %) and anhydrous *N,N′*-dimethylformamide (DMF) packed under nitrogen, and sodium hydride (NaH) were obtained from Fisher Scientific (UK). NaH oil powder was washed with n-hexane to remove the coated oil of the powder.

Step 1: *production of N,N′-Bis-(carbethoxymethyl)-N,N′-diacetyl-1, 4-phenylenediamine*

First, *N,N′*-Diacetyl-1,4-phenylenediamine (compound 1, figure S1) (5 g, 26 mmol) was dissolved in 100 mL dimethyl sulfoxide (DMF) under argon with constant stirring for 5 min. 1.25 g (52 mmol) of sodium hydride (NaH) were added to the solution. The mixture was heated up to 100 °C for 30 min, where the purple suspension became pale coloured. The solution was cooled down in ice bath and 7.5 mL (8.7 g, 70 mmol) of ethyl chloroacetate ($ClCH_2CO_2CH_2CH_3$) were added dropwise during vigorous stirring. The thick suspension was dissolved upon the addition of ethyl chloroacetate. The DMF solvent was evaporated under reduced pressure to give the crude materials. Subsequently, 200 mL dichloromethane (CH_2Cl_2) were added to the above crude materials and the precipitate was filtered off. The solution of dichloromethane containing a mixture of the products was evaporated to dryness. The crude materials were dissolved in 5 mL of dichloromethane. The solution was loaded into a puriFlash® (High Performance HP) column (size 80 g) using puriFlash® system 350 (Advion, Interchim/France), eluted with dichloromethane and n-hexane in a 1:2 ratio and then with 10 % methanol and dichloromethane. The pure product, *N,N′*-Bis-(carbethoxymethyl)-*N,N′*-diacetyl-1,4-phenylenediamine or diethyl 2,2'-(1,4-phenylenebis(acetylazanediyl)) diacetate (compound 2, figure S1) was obtained as an off-white solid with a 25 % yield.

Step 2: *N,N′ -Bis-(carboxymethyl)-N,N′ -dinitroso-1,4-phenylenediamine*

Compound 2 (1 g, 3.2 mmol) was dissolved in 10 % H_2SO_4 solution (30 mL) and stirred under argon for 10–30 min. The solution was refluxed for 2 h where the ester groups were hydrolysed and the suspension of compound 2 was dissolved. The resulting solution of *N,N′*-Bis-(carbethoxymethyl)-1,4-phenylenediamine (compound 3, figure S1) was then cooled down in an ice bath. Sodium nitrite ($NaNO_2$, 20 mL of 0.3 M) was prepared, degassed with argon gas and added to the above solution, releasing a pink coloured gas and then turning instantly to a yellow pale solution. The solid product started to precipitate while the solution was in the ice bath. The precipitate was filtered and washed several times with ice water to remove any traces of the acid. The product, *N,N′*-bis-(carboxymethyl)-*N,N′*-dinitroso-1,4-phenylenediamine (compound 4, figure S1), was dried under vacuum in the dark yielding 0.64 g (2.3 mmol). The spectroscopic properties of the final synthesised compound were identical to those reported in the literature; UV–Vis in 25 mM phosphate buffer, pH = 7.4: (λ_{max} 300

nm, 13.5 mM^{-1} cm^{-1}) ; (λ_{max} 217 nm, 13 mM^{-1} cm^{-1}) and ^{1}H NMR (δ, m, in D_2O/pH 7.55 phosphate buffer): 4.66, s, 4H of RN(NO)(CH_2)COO− ; 7.70, s, 4H of Ph ring (Namiki *et al.*, 1997).

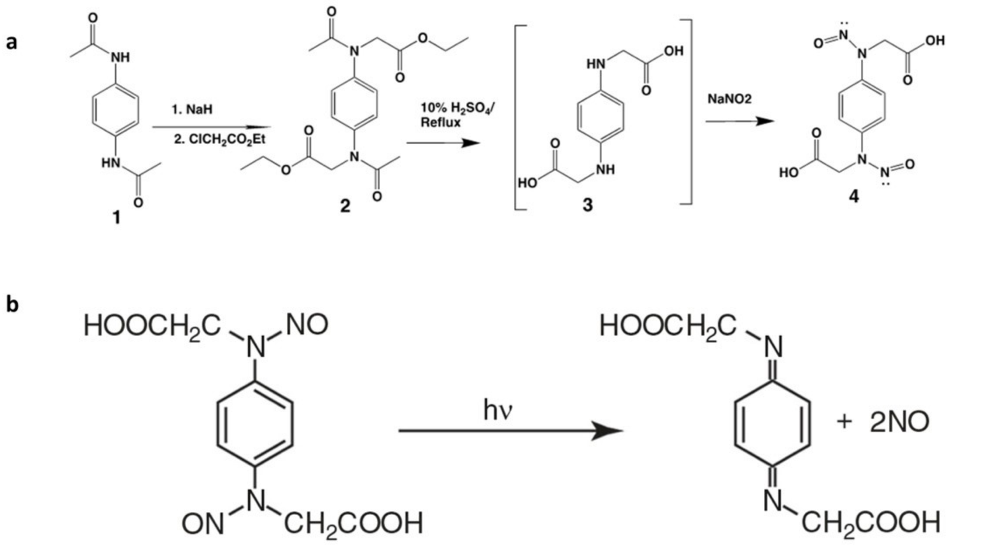

Figure S1 (a) Synthesis of *N*,*N′*-bis-(carboxymethyl)-*N*,*N′*-dinitroso-1,4-phenylenediamine (compound 4) or NO cage. (b) The photolysis reaction to release two NO molecules per photocage molecule (Tosha *et al.*, 2017).

S2. Crystal growth

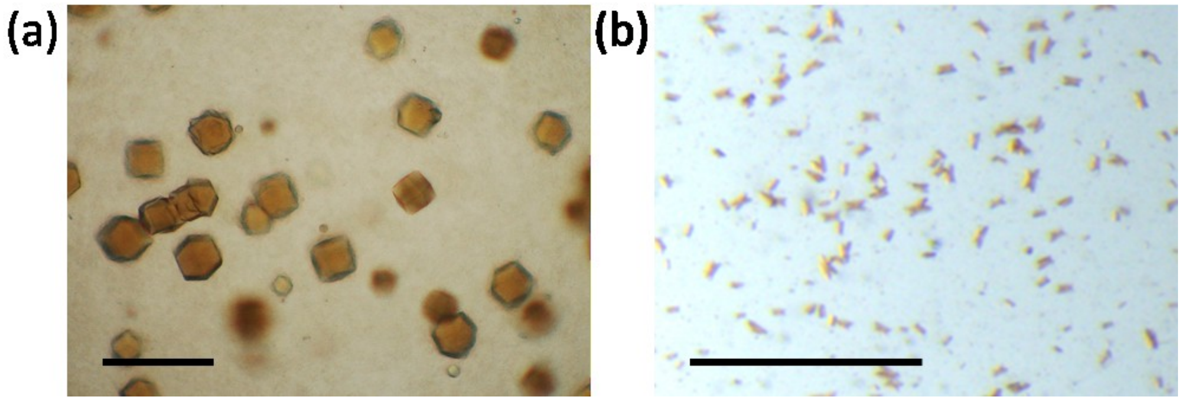

Figure S2 Microcrystals in mother liquor from batch crystallisation experiments of (a) McCP-β and (b) DtpB. Typical crystal sizes were 30 µm (McCP-β) and 10 µm (DtpB). Scale bars represent 100 µm.

S3. Optical and X-ray scattering properties of thin films

Various thin films were tested for their optical properties and suitability for the time-resolved experiments using UV laser photoactivation. UV-Vis absorbance spectra (figure S3) were collected using a microspectrophotometer developed at beamline I24 for on-line and *in crystallo* optical spectroscopy. The setup features two off-axis reflective objectives, a Shamrock 303i (Andor Technology) spectrometer, a Newton EM CCD detector and a fibre-coupled Xenon light source (Thorlabs) with a continuous spectrum over the wavelength range of 250–800 nm. Single layers of 6 µm Mylar (SPEX SamplePrep), 12.5 µm EVAL (Kuraray) and 12.5 µm polyvinylidene chloride (PVDC) were mounted at the focal point of the two objectives. The spectra were an accumulation of 50 exposures each of 10 ms duration. The diameter of the white light beam was approximately 50 µm. Data were processed and plotted with OriginPro using a Savitzky-Golay filter to remove noise.

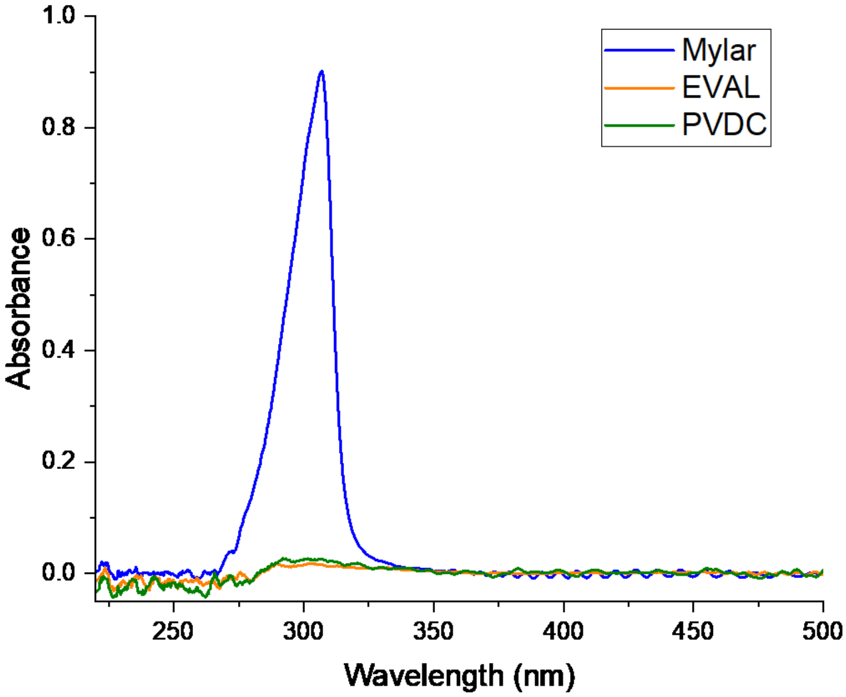

Figure S3 UV-Vis absorbance spectra for three thin films used for sealing fixed targets: 6 µm Mylar, 12.5 µm EVAL, and 12.5 µm PVDC.

In comparison to mylar which we typically use for sealing fixed target chips, EVAL has increased optical transmission at 308 nm (figure S3) at the expense of increased X-ray scattering, resulting in increased background on the detector (figure S4). X-ray background scattering from the thin films was compared by collecting a series of exposures from two layers of film in a typical fixed target configuration. Images were collected with 10 ms exposures of a 20 × 20 µm beam at 20 keV, with the

DECTRIS EIGER2 X CdTe 9M detector at 320 mm distance from the sample position. Figure S4a is summed from 100 images for each film and air, with resolution rings indicating the location of the diffuse scattering rings. Figure S4b shows counts as a function of resolution, averaged from 100 images from the three sampled films and air. As shown in both panels of figure S4, Mylar has the lowest background scatter, featuring a low intensity diffusive ring at approximately 6 Å and is confirmed to be appropriate for serial experiments using fixed targets. EVAL features a high intensity peak at 4.5 Å, while PVDC has several diffusive peaks across a resolution range of 6–2 Å making it a poor choice compared to EVAL, even though the optical transmission of EVAL and PVDC as similar at 308 nm.

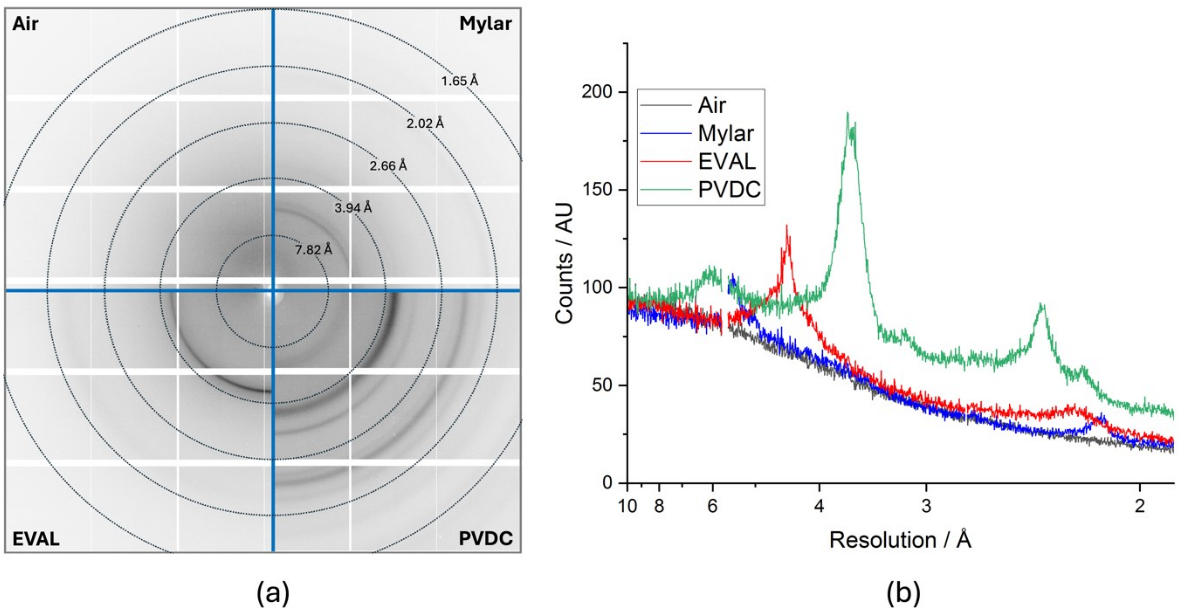

Figure S4 Comparison of diffuse X-ray scatter at 20 keV for air and three thin films (6 µm Mylar, 12.5 µm EVAL and 12.5 µm PVDC) used for sealing fixed targets. (a) Stack of 100 images recorded by the detector for each film and air. (b) Detector counts as a function of resolution, from the same 100 frames.

S4. Fixed target hardware at SACLA and triggering schemes

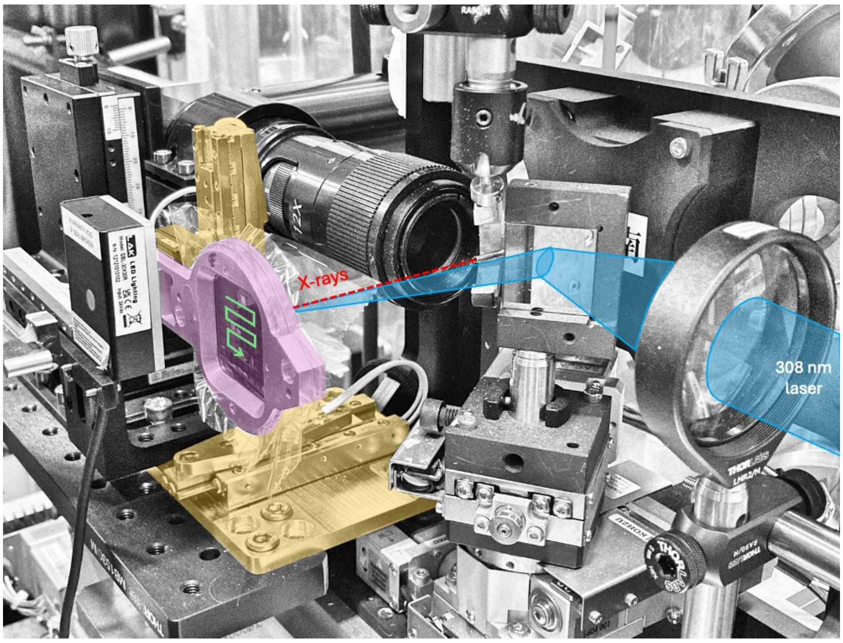

Figure S5 Fixed target hardware in place at SACLA with chip highlighted in pink, motion stages in gold, and laser light path shown in blue. Minimal differences between the experimental setup at Diamond and SACLA enable straightforward comparison of results from the two X-ray sources.

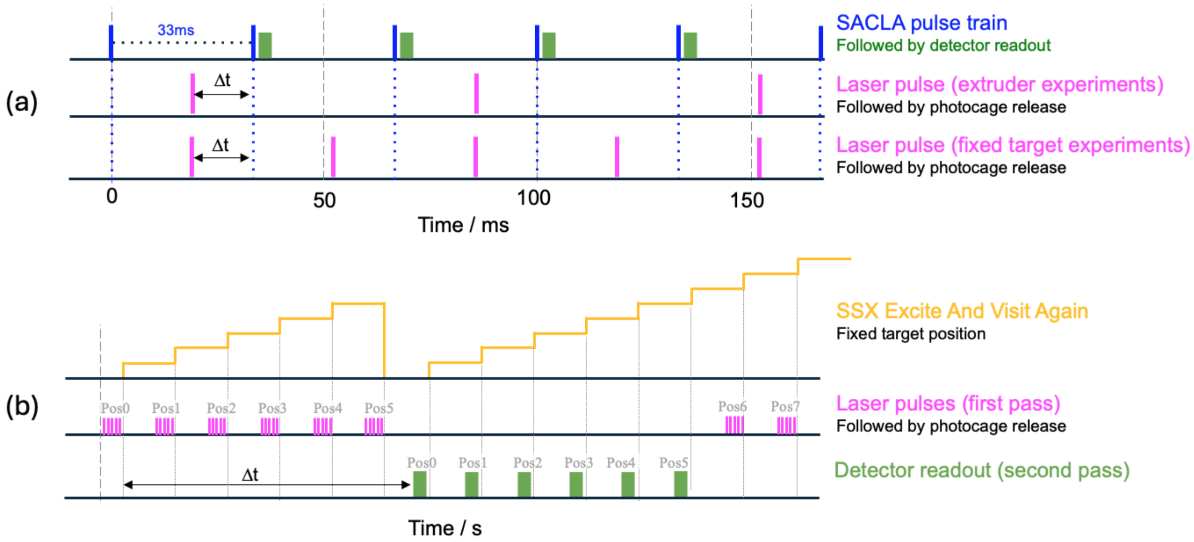

Figure S6 Triggering schemes used at SACLA and Diamond. (a) shows the triggering schemes for extruder and fixed target SFX which followed a standard pump-probe scheme. (b) illustrates timings for laser excitation and X-ray data collection for the excite and visit again strategy employed for SSX. A 6 unit repeat is shown for illustrative purposes: the fixed targets employed utilise a 40 unit repeat.

S5. Comparison of room temperature serial crystallography and 100 K single-crystal structures of McCP-β

The first room temperature crystal structure of McCP-β was determined to 1.80 Å resolution by fixed target SFX at SACLA. This damage-free structure shows close similarity to the previously published cryogenic single-crystal structure (Adams *et al.*, 2019), with the differences being isolated to side chain movements in the loops between β-strands, and at the heme site (figure S7a, b). These structural differences may be X-ray induced or may reflect the changing energy landscape of the protein at different temperatures, with different conformations being favoured (Keedy *et al.*, 2014). Despite the protein being a homodimer, the NO exhibits different conformations between chains so figure S7 shows both chains A and B. Compared to 100 K, at room temperature the Phe 32 side chain in the heme site is displaced away from the iron, with the Cζ atom moving by 1.1 Å and 0.9 Å in chains A and B respectively. Phe 32 occupies a position at the entrance to the distal heme site, therefore the flexibility of Phe 32 is necessary for the NO binding function of the protein. Phe 61 does not sit at the entrance to the distal heme site, therefore does not have the conformational flexibility required by Phe 32. This interpretation is supported by the higher B-factors of the atoms in the Phe 32 side chain, averaging 41 $Å^2$ in the room temperature structure, compared with 22 $Å^2$ in Phe 61 indicating the flexibility of the residue.

An NO-bound structure of McCP-β, obtained by soaking with PROLI NONOate, was also determined at room temperature (1.85 Å resolution) and this is compared to the equivalent cryogenic structure (Adams *et al.*, 2023) in figure S7c,d. The differences between structures determined at the two temperatures are small, as in the resting state. Both distal phenylalanine residues adopt the same rotamer, although the phenyl group of Tyr 131 is rotated by approximately 15° around its axis in both chains. Although the side chain does not interact with the heme, the carboxyl group of the residue stabilises the proximal His 123 via a hydrogen bond. This stabilisation of the proximal histidine residue in its position bound to the Fe may be responsible for the lack of conversion to a proximally bound NO as seen in other cytochromes *c′* (Kekilli *et al.*, 2017). In the cryogenic structure, determined to 1.56 Å resolution, the NO is modelled in two orientations in chain B (figure S7d, magenta). The Fe–N bond length and Fe–N–O bond angles between the two copies are similar, but the NO is oriented in two opposing directions. This second orientation is not seen in any of the room temperature structures collected here, although the lower resolutions (1.75–2.25 Å) of these structures could obscure multiple orientations of the NO.

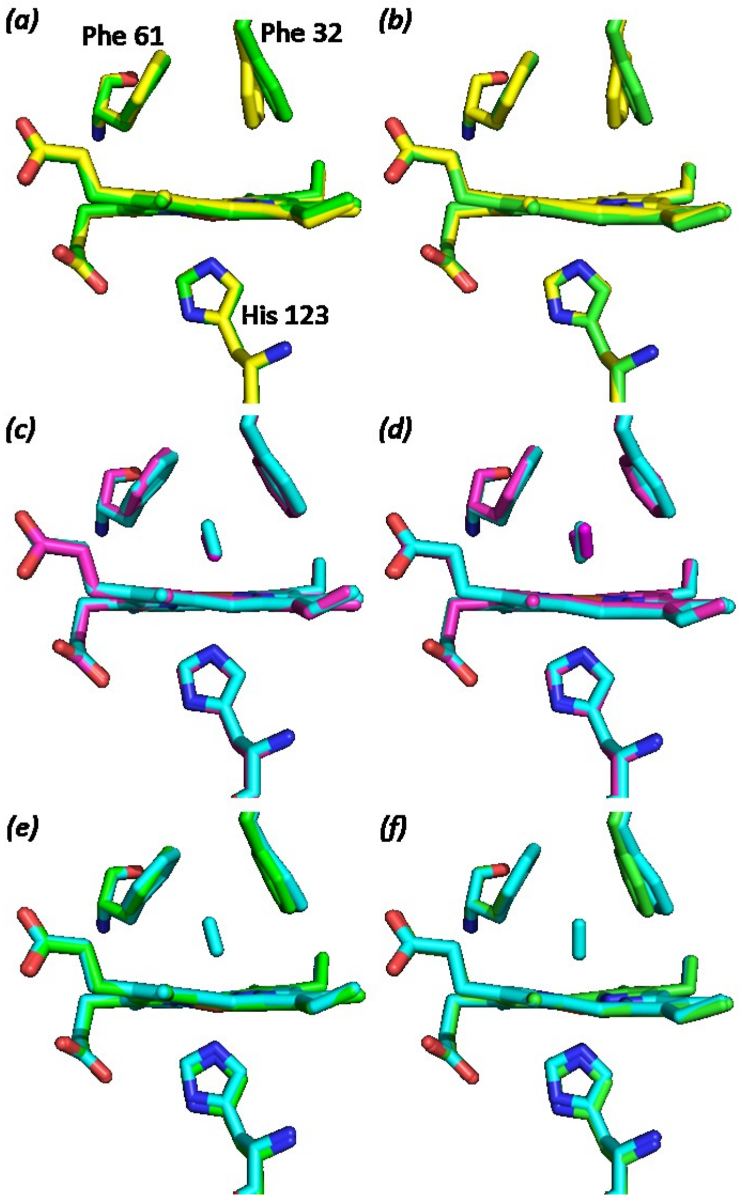

Figure S7 Comparison of the heme site of McCP-β in structures determined by rotation crystallography at 100 K (Adams et al., 2019, 2023), and by room temperature serial crystallography. Panels a and b show chain A and chain B resting state structures respectively with the cryogenic model (6HIH) in yellow and SFX room temperature model (9HQT) in green. At room temperature, the distal Phe 32 residue is displaced further from the heme. Panels c and d show chain A and B NO-bound structures, soaked with PROLI NONOate as the NO donor. The cryogenic model (7ZPS) is magenta while the SFX room temperature model (9HS8) is cyan. Panels e and f compare chains A and B of the room temperature serial structures with and without NO bound, in cyan and green respectively.

S6. 1600 µJ NO cage structure of McCP-β

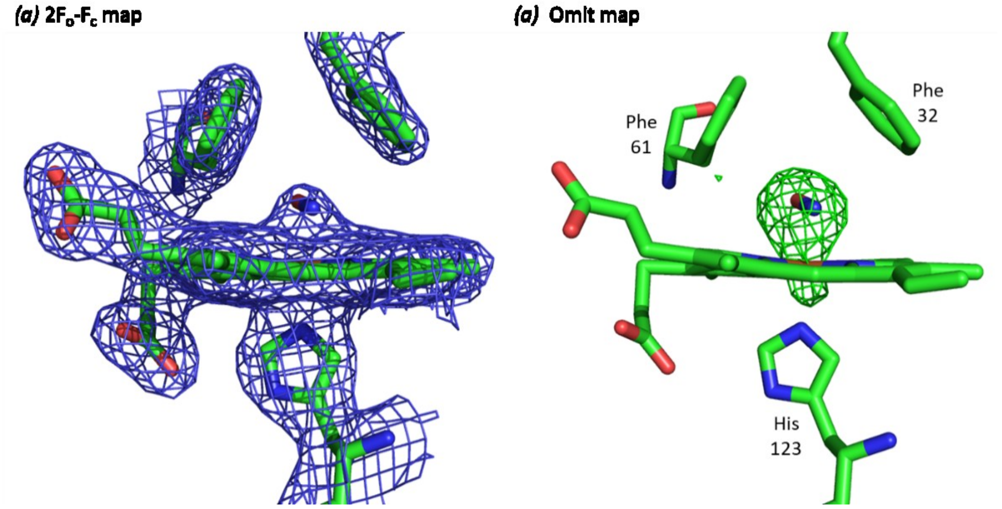

Figure S8 High laser intensity illumination (1600 mJ, PDB: 9HTC) McCP-β maps and chain A, $2F_o$–F_c (*a*) and omit (*b*), contoured at 1σ and 5σ respectively. In this experiment, the fixed target chip was reversed in orientation compared to the other structures in this manuscript, and Mylar film was used to seal the chip, with the result that far less light reached the sample.

S7. DtpB resting state structure

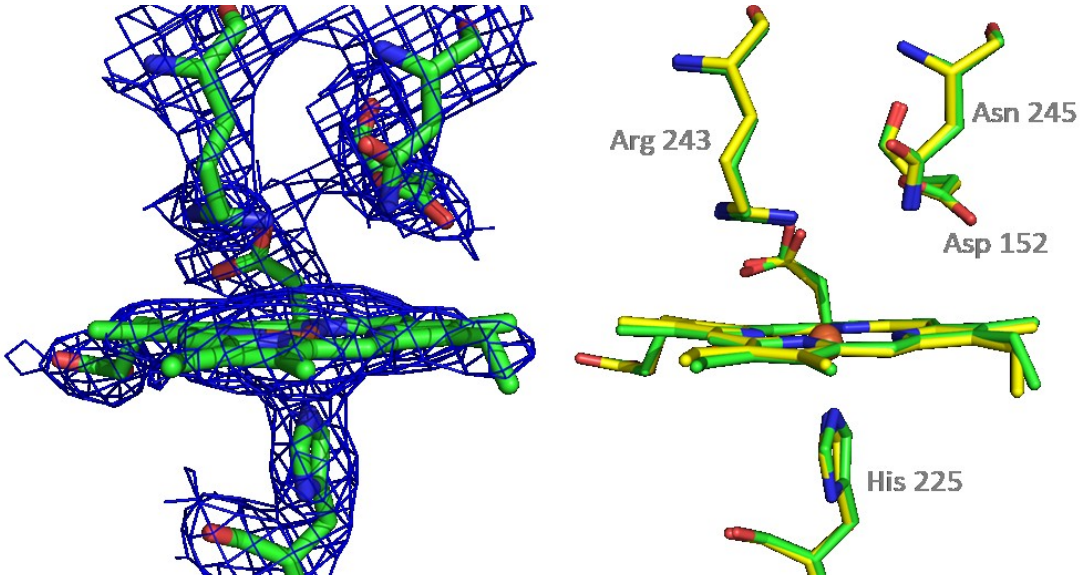

Figure S9 SSX resting state structures of DtpB, chain A. (a) $2F_o$–F_c map, contoured at 1σ. (b) Comparison of the SSX structure (green) and previously published SFX structure (yellow). No major differences are seen between the two structures.

S8. B-factors of refined structure against NO occupancy

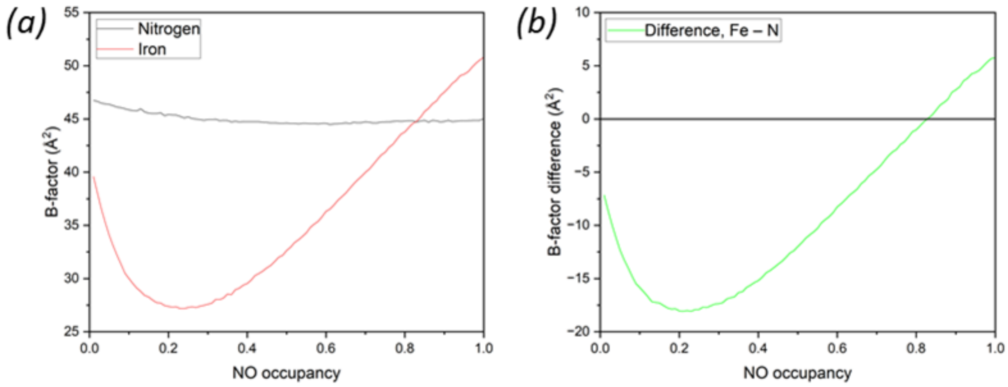

Figure S10(a) B-factors of the haem iron (red) and the NO nitrogen (black) atoms from chain A of the DtpB structure with 64.4 μJ laser power, plotted against the fixed NO occupancy at which the structure was refined. (b) Difference between nitrogen and iron B-factors shown in (a), green. The occupancy is taken as where the difference in B-factors is zero, in this example at 0.84.

S9. Data processing and refinement statistics

Table S1 Data processing and refinement statistics for McCP-β structures, including resting states collected at SACLA and I24, time-resolved NO-bound structures collected using a photocage, a negative control photocage structure, and an NO-bound structure soaked with PROLI NONOate. Values in parentheses refer to the outermost resolution shell. For all data the space-group was $P2_13$.

Dataset	Resting state SFX	Resting state SSX	PROLI NONOate	Dark negative control	1600 µJ laser excitation	9.5 µJ laser excitation	0.95 µJ laser excitation	0.19 µJ laser excitation	12.9 µJ laser excitation SFX
Resolution (Å)	33.89−1.80 (1.83−1.80)	75.82–2.15 (2.19–2.15)	76.09−1.85 (1.88−1.85)	76.00–2.20 (2.24–2.20)	76.09–1.80 (1.83−1.80)	75.91–2.00 (2.03–2.00)	75.95–1.80 (1.83–1.80)	43.83–1.75 (1.78–1.75)	35.80-2.25 (2.29–2.25)
Unit cell (Å) *a* = *b* = *c*	107.1	107.2	107.4	107.3	107.4	107.2	107.4	107.3	107.8
Merged crystals	14 394	6692	11 352	4368	11 352	6005	5857	14 324	11 235
Unique reflections	38 160 (3657)	22 647 (1156)	35 508 (1741)	21 205 (1049)	38 525 (1922)	28 000 (1378)	38 471 (1909)	41 713 (2043)	20 088 (1013)
Completeness	100.0 (100.0)	100.0 (100.0)	100.0 (100.0)	100.0 (100.0)	100.0 (100.0)	100.0 (100.0)	100.0 (100.0)	100.0 (100.0)	100.0 (100.0)
Multiplicity	222.0 (152.9)	69.9 (58.1)	175.4 (137.3)	40.0 (34.0)	171.8 (127.2)	63.0 (48.8)	59.7 (47.1)	121.2 (85.7)	430.4 (356.0)
$CC_{1/2}$	0.940 (0.532)	0.977 (0.305)	0.998 (0.534)	0.974 (0.373)	0.998 (0.291)	0.992 (0.366)	0.993 (0.399)	0.997 (0.294)	0.996 (0.358)
R_{split}	0.208 (0.783)	0.188 (1.017)	0.068 (0.936)	0.184 (0.753)	0.070 (1.234)	0.122 (1.042)	0.111 (0.943)	0.076 (1.270)	0.109 (1.160)
I/σ(I)	3.7 (1.3)	7.7 (1.2)	11.0 (0.9)	9.0 (1.7)	10.2 (0.7)	5.8 (0.7)	6.3 (0.7)	8.2 (0.5)	6.3 (0.6)
N_{obs}	14 465 627 (498 904)	1 583 238 (67 218)	6 227 273 (238 956)	848 796 (35 661)	6 617 131 (244 570)	1 764 785 (67 297)	2 295 621 (89 824)	5 054 526 (175 036)	8 646 734 (360 601)
R_{work}	0.190	0.196	0.155	0.192	0.161	0.162	0.161	0.167	0.190
R_{free}	0.219	0.237	0.180	0.231	0.195	0.194	0.182	0.199	0.223

RMSD bond length (Å)	0.0173	0.0116	0.0128	0.0050	0.0119	0.0099	0.0122	0.0117	0.0107
RMSD bond angles (°)	2.317	1.667	2.553	2.112	2.514	2.494	2.624	2.528	1.927
Ramachandran favoured (%)	97.40	97.03	96.65	98.51	96.64	96.64	97.77	97.39	95.90
PDB code	9HQT	9HU1	9HS8	9HYZ	9HTC	9Q86	9HTV	9HTT	9QME

Table S2 Data processing and refinement statistics for DtpB NO-bound SSX structures obtained using different laser energies, and an apo structure without NO cage. Values in parentheses refer to the outermost resolution shell. *Structures at 16.1 µJ and 8.05 µJ are less reliable due to the low numbers of crystals merged in the datasets. For all data the space-group was $P2_12_12_1$.

Dataset	64.4 μJ	32.2 μJ	16.1 μJ*	8.05 μJ*	0.81 μJ	Resting state
Resolution (Å)	79.75–2.40 (2.44–2.40)	76.87–2.40 (2.44–2.40)	79.71–2.40 (2.44–2.40)	79.66–2.40 (2.44–2.40)	79.71–2.40 (2.44–2.40)	103.66–2.75 (2.80–2.75)
Unit cell (Å), a, b, c	87.2, 123.2, 195.5	86.7, 121.4, 198.2	87.1, 123.0, 195.4	87.1, 122.9, 195.0	87.1, 122.9, 195.6	86.4, 121.4, 199.3
Merged crystals	6870	5536	3612	3109	5003	5895
Unique reflections	83 011 (4087)	82 452 (4038)	82 830 (4084)	82 535 (4074)	82 839 (4089)	55 273 (2716)
Completeness (%)	100.0 (100.0)	100.0 (100.0)	100.0 (100.0)	100.0 (100.0)	100.0 (100.0)	100.0 (100.0)
Multiplicity	39.4 (30.2)	34.6 (25.3)	23.3 (17.4)	20.7 (15.2)	28.8 (22.1)	22.2 (19.2)
$CC_{1/2}$	0.941 (0.264)	0.943 (0.586)	0.945 (0.312)	0.943 (0.390)	0.954 (0.397)	0.916 (0.297)
R_{split}	0.258 (1.391)	0.191 (0.583)	0.23 3(1.123)	0.205 (0.862)	0.211 (0.901)	0.303 (1.018)
$I/\sigma(I)$	4.5 (0.9)	5.8 (1.6)	4.5 (1.0)	5.4 (1.3)	4.9 (1.2)	5.1 (1.3)
N_{obs}	3 266 484 (123 533)	2 852 879 (102 088)	1 933 063 (70 886)	1 711 751 (62 087)	2 384 569 (90 259)	1 225 800 (52 176)
R_{work}	0.188	0.182	0.193	0.177	0.178	0.191
R_{free}	0.214	0.223	0.212	0.220	0.226	0.244
RMSD bond lengths (Å)	0.0035	0.0024	0.0027	0.0025	0.0024	0.0027
RMSD bond angles (°)	1.217	0.983	1.065	1.038	1.015	1.119
Ramachandran favoured (%)	97.69	97.92	98.30	97.70	97.48	94.62
PDB code	9I4S	9I4U	9I6G	9IA9	9IAA	9I4Q

Table S3 Data processing and refinement statistics for DtpB NO-bound SFX structures obtained at different timepoints and laser energies. Values in parentheses refer to the outermost resolution shell. For all data the space-group was $P2_12_12_1$.

All	10 μJ 10 ms	30 μJ 10 ms	30 μJ 100 μs	100 μJ 100 μs
Resolution range (Å)	103.95–1.56 (1.59–1.56)	104.11–1.69 (1.72–1.69)	104.64–1.52 (1.55–1.52)	104.67–1.67 (1.70–1.67)
Unit cell (Å) *a,b,c*	87.07, 122.88, 194.94	87.17, 123.06, 195.85	87.47, 124.39, 193.51	87.49, 124.44, 193.56
Merged crystals	20 229	9381	19408	5038
Total reflections	49 890 133 (1 968 424)	22 264 669 (873 001)	53 882 611 (2143549)	13 024 025 (503747)
Unique reflections	295 889 (14 630)	234 283 (11 573)	322 685 (15959)	244 152 (12 054)
Multiplicity	168.6 (134.5)	95.0 (75.4)	167.0 (134.3)	53.3 (41.8)
Completeness (%)	100 (100)	100 (100)	100 (100)	100 (100)
I/σ(I)	18.1 (0.9)	15.04 (1.0)	19.4 (0.9)	13.4 (0.8)
R_{split}	0.096 (1.256)	0.136 (1.252)	0.089 (1.224)	0.195 (1.468)
CC1/2	0.995 (0.322)	0.988 (0.314)	0.996 (0.317)	0.969 (0.238)
R_{work}	0.209	0.190	0.209	0.216
R_{free}	0.234	0.219	0.235	0.244
RMSD bond lengths (Å)	0.0116	0.0132	0.0155	0.0147
RMSD bond angles (°)	1.980	2.173	1.954	2.282
Ramachandran favoured (%)	100.00	97.81	96.16	95.83
PDB code	9HL1	9HO7	9HXX	9HYV

Table S4 NO occupancies, as determined by three different methods, in DtpB SSX structures from a laser power titration. Values represent an average of those determined for the six chains in the asymmetric unit. The values from multicopy refinement were chosen for the final structures, shown in figure 5.

Laser power (µJ)	B-factor comparison	On-the fly / F_o–F_c peak height	Multicopy refinement
64.4	0.84	0.77	0.71
32.2	0.58	0.56	0.60
16.1	0.65	0.56	0.47
8.05	0.50	0.41	0.29
0.81	0.39	0.34	0.25
